# Regulatory and effector T cell subsets in tumor-draining lymph nodes of patients with squamous cell carcinoma of head and neck

**DOI:** 10.1186/s12865-022-00530-3

**Published:** 2022-11-15

**Authors:** Marzieh Norouzian, Fereshteh Mehdipour, Mohammad Javad Ashraf, Bijan Khademi, Abbas Ghaderi

**Affiliations:** 1 Department of Laboratory Sciences, School of Allied Medical Sciences, Hormozgan University of Medical Sciences, Bandar Abbas, Iran; 2 Shiraz Institute for Cancer Research, School of Medicine, Shiraz University of Medical Sciences, P.O. Box: 71345-3119, Shiraz, Iran; 3 Department of Oral Pathology, School of Medicine, Shiraz University of Medical Sciences, Shiraz, Iran; 4 Department of Otolaryngology, School of Medicine, Shiraz University of Medical Sciences, Shiraz, Iran

**Keywords:** Head and neck squamous cell carcinoma, Tumor draining lymph nodes, T lymphocytes, Regulatory T cells, Cytokine profile, Immune profile

## Abstract

**Background:**

A crucial role for the immune system has been proposed in the establishment and progression of head and neck squamous cell carcinoma (HNSCC). In this study, we investigated the cytokine and regulatory profiles of T cells in tumor draining lymph nodes (TDLNs) of patients with HNSCC.

**Results:**

The frequencies of CD4^+^TNF-α^+^ and CD4^+^TNF-α^hi^ negatively were associated with poor prognostic factors such as LN involvement (*P* = 0.015 and *P* = 0.019, respectively), stage of the disease (*P* = 0.032 and *P* = 0.010, respectively) and tumor size (*P* = 0.026 and *P* = 0.032, respectively). Frequencies of CD8^+^IFN-γ^+^ and CD8^+^IFN-γ^+^ TNF-α^+^ T cells showed negative relationship with tumor grade (*P* = 0.035 and *P* = 0.043, respectively). While, the frequencies of CD4^+^IL-4^+^, CD8^+^IL-10^+^, CD8^+^IL-4^+^T cells were higher in advanced stages of the disease (*P* = 0.042, *P* = 0.041 and *P* = 0.030, respectively) and CD4^+^IFN-γ^+^TNF-α^−^, CD8^+^IL-4^+^ and CD8^+^IFN-γ^+^TNF-α^−^ T cells were higher in patients with larger tumor size (*P* = 0.026 and *P* = 0.032, respectively). Negative associations were found between the frequencies of CD4^+^CD25^+^Foxp3^+^ and CD4^+^CD25^+^Foxp3^+^CD127^low/−^ Treg cells and cancer stage (*P* = 0.015 and *P* = 0.059).

**Conclusion:**

This study shed more lights on the changes in immune profile of T cells in TDLNs of HNSCC. Larger tumor size and/or LN involvement were associated with lower frequencies of CD4^+^TNF-α^+^, CD8^+^IFN-γ^+^ and CD8^+^IFN-γ^+^TNF-α^+^ but higher frequency of CD4^+^IL-4^+^ T cells. Moreover, Foxp3^+^Tregs correlated with good prognostic indicators.

**Supplementary Information:**

The online version contains supplementary material available at 10.1186/s12865-022-00530-3.

## Introduction

Head and neck squamous cell carcinoma (HNSCC) is one of the most common malignancies with the annual global incidence of 400,000 cases and accounts for 1–2% of all cancer-related deaths. The essential role of the immune response against tumors in the head-neck region was reported in the 1970s when infiltration of T cells at tumor sites was shown to have a correlation with prognosis in oral squamous cell carcinoma (OSCC) [1]. It is now evident that both cancer progression and resistance to therapy are affected by the interaction between tumor cells and their surrounding microenvironment [2, 3]. Although HNSCC microenvironment is highly infiltrated by immune cells, these highly immune-modulatory tumors are believed to evade the immune surveillance through different mechanisms which are largely unknown and controversial [4–7]. An in depth understanding of these mechanisms can lead to future cancer therapy based on modulating interactions between tumor cells and the immune system.

Little is known about the influence of tumor-immune cells interactions on the activation and/or regulation of antitumor cellular immune responses in HNSCC. Further work is still needed to find the effect of neoplastic cells on the function of important immune cells such as T CD4^+^ and CD8^+^ lymphocytes. CD4^+^ and CD8^+^ populations are two major classes of T cells. Various subpopulations in both CD4^+^ and CD8^+^ T cells have been identified which have different functions. CD4^+^ T cells are subdivided into different subsets: T helper 1 (Th1), Th2, Th9, Th17, Th22, Treg, and T follicular helper cells (Tfh) based on their transcription factors and signature cytokines. Each Th subset with specific cytokine profile can mediate protective or pathogenic function [8]. Similar to subsets of CD4^+^ T cells, CD8^+^ T cells or cytotoxic T cells (Tc), under certain circumstances, express interferon-γ (IFN-γ) (Tc1), IL-4/IL-5/IL-13 (Tc2), IL-9 (Tc9), IL-17 (Tc17) or suppressive activity [9].

Mouse and human studies supported the importance of immune responses in HNSCC; in animal models of chemically induced tongue cancer, reduction in T cell function resulted in tumor development or recurrence [10], indicating that adaptive immunity plays a pivotal role in HNSCC. Recent studies have shown changes in immune responses in patients with HNSCC [11–13]. Kesselring et al. reported the changes in the phenotype of Th17 cells characterized by downregulation of CD161 receptor in peripheral blood and metastatic lymph nodes in patients with HNSCC [11]. Another investigation showed the presence of functionally activated regulatory T cells along with lower frequency of Th1 cells in peripheral blood and tumor microenvironment in patients with laryngeal squamous cell carcinoma [13].

Although, the presence of certain immune cells in the tumor microenvironment is related to favorable outcome [14], there are studies which propose that antitumor immune responses are impaired in HNSCC and contribute to disease progression [15]. It has been revealed that in the microenvironment of HNSCC, increased expressions of IL-4, IL-10 and transforming growth factor β (TGF-β) suppress adaptive immunity and promote tumor escape from immune system [16, 17]. Functional disorders in circulating and tumor-infiltrating T lymphocytes, impaired NK cell anti-tumor activity and elevated levels of Tregs as well as a general decrease in lymphocyte counts were demonstrated in patients with HNSCC [18].

During the early stages of most cancers, tumor draining lymph nodes (TDLNs) are the initial sites in which tumor specific immune cells respond to tumor antigens [19, 20]. The existence of tumor-specific effector and suppressive T cells has been shown in TDLNs [21]. It has been suggested that the outcome of T cell responses to tumor is dependent on the delicate balance between effector and regulatory T cells [22]. Tumor cell products would have an effect on cellular composition of lymph nodes that support tumor cells growth [23–25]. Recent researches indicated the association between immune cell compounds in TDLNs and cancer prognosis [26, 27]. Therefore, in this study, we determined the cytokine profile of CD4^+^ and CD8^+^ T cells as well as the frequency of Foxp3^+^ Treg cells in the TDLNs of HNSCC and assessed their association with cancer parameters.

## Results

### Frequencies of cytokine-producing CD4^+^ and CD8^+^ T cells in the TDLNs of patients with HNSCC (tongue and laryngeal SCC)

**Fig. 1:**
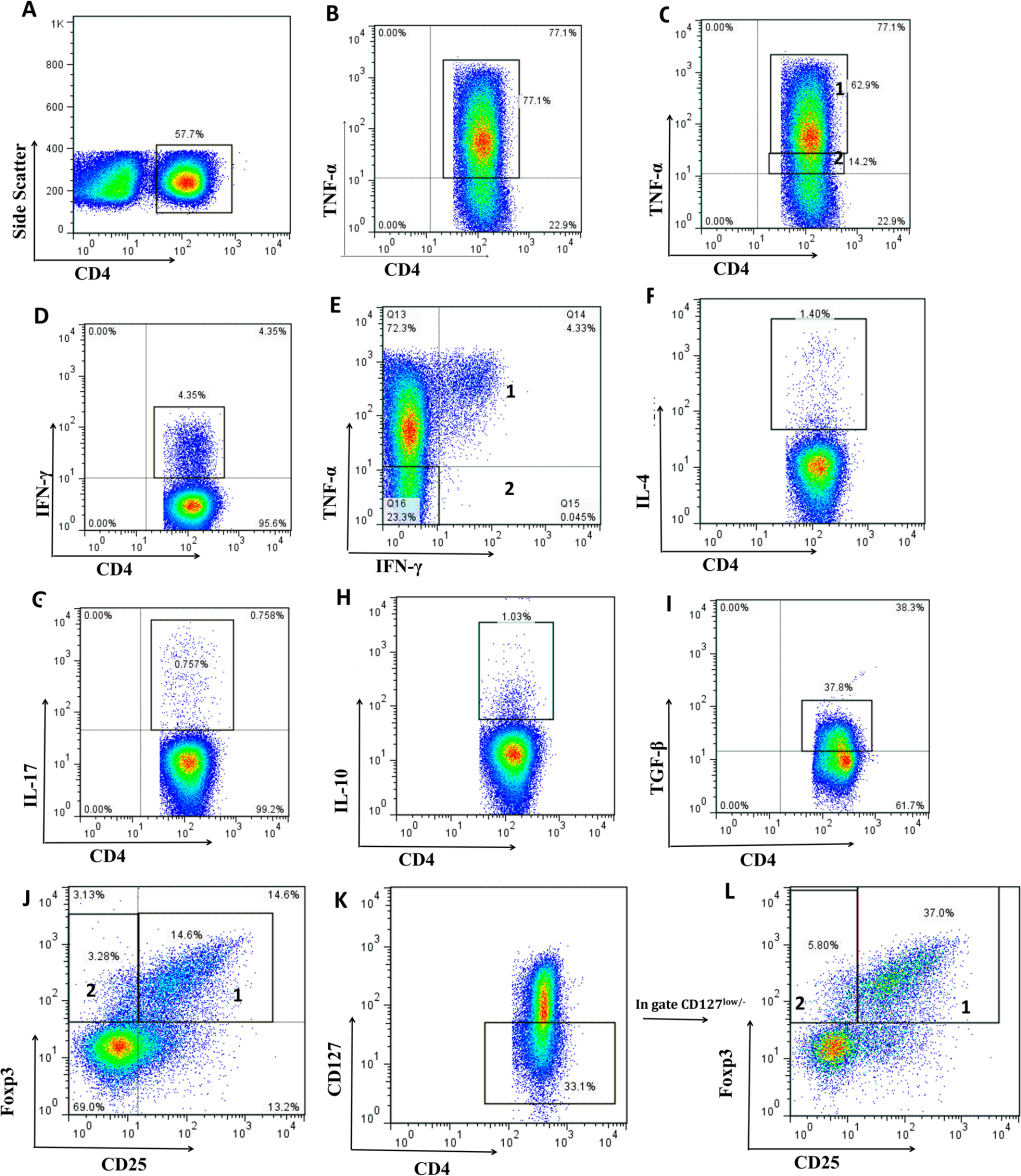
Flow cytometry analysis of CD4^+^ T cell subsets in the tumor draining lymph nodes of patients with HNSCC. Lymphocytes were gated **A** CD4^+^ T cells were gated followed by defining these subpopulations in CD4^+^ T cells gate: **B** TNF-α^+^ cells, **C** (1)TNF-αhi cells, (2) TNF-αlow cells, **D** IFN-γ^+^ cells, **E** (1) IFN-γ^+^TNF-α^+^ cells, (2) IFN-γ^+^TNF-α^−^ cells, **F** IL-4^+^ cells, **G** IL-17^+^ cells, **H** IL-10^+^ cells, **I** TGF-β^+^ cells subsets according to their cognate cytokine expression and **J** (1)CD25^+^Foxp3^+^ cells, (2) CD25^−^Foxp3^+^ cells, **K** CD127 ^low/−^ cells subsets according to CD25, Foxp3 and CD127 expression and **L** (1) CD25^+^Foxp3^+^ cells, (2) CD25^−^Foxp3^+^ cells in CD127^low/−^ cells gate

**Fig. 2:**
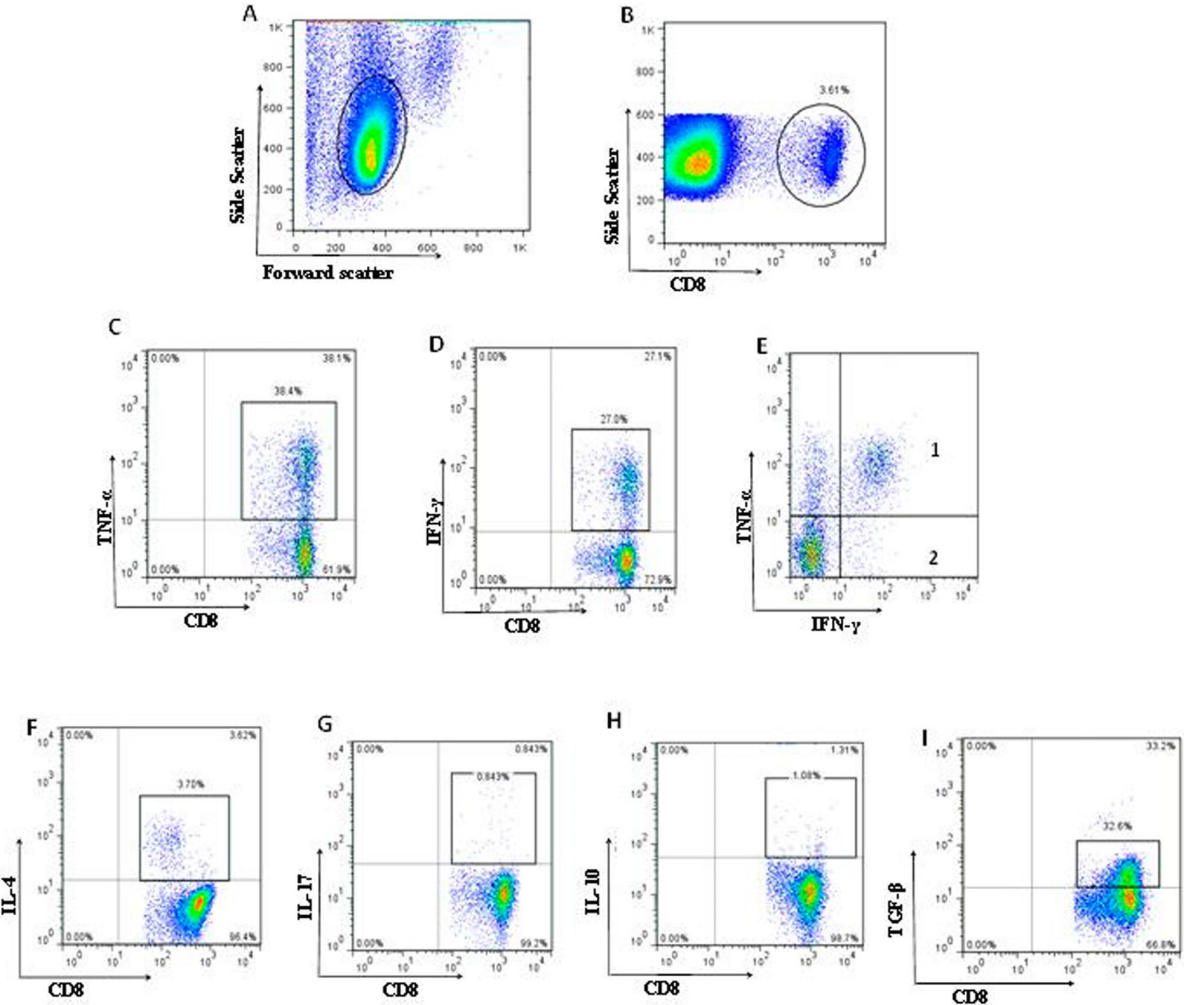
Flow cytometry analysis of CD8^+^ T cell subsets in the tumor draining lymph nodes of patients with HNSCC. **A** Lymphocytes were gated, **B** CD8^+^ T cells were gated followed by defining these subpopulations in CD8^+^ T cells gate: **C** TNF-α^+^ cells, **D** IFN-γ^+^ cells, **E** (1) IFN-γ^+^TNF-α^+^ cells, (2) IFN-γ^+^TNF-α^−^ cells, **F** IL-4^+^ cells, **G** IL-17^+^ cells, **H** IL-10^+^ cells, **I** TGF-β^+^ cells subsets according to their cognate cytokine expression

The frequencies of CD4^+^ and CD8^+^ T cells were determined in the lymphocyte gate in patients with HNSCC and the percentages of different cytokine-producing subsets of CD4^+^ and CD8^+^ T lymphocytes including IFN-γ^+^, TNF-α^+^, IL-4^+^, IL-17^+^, IL-10^+^ and TGF-β^+^ T cells were assessed (Figs. 1, 2 and Table 1, unstimulated cells served as controls; see Additional file 1: Figures S1 and S2). Moreover, the frequencies of CD25^+^Foxp3^+^, CD25^−^Foxp3^+^, CD25^+^Foxp3^+^CD127^low/−^ and CD25^−^Foxp3^+^CD127^low/−^ cells were assessed in CD4^+^ T cell gate (Fig. 1, Table 1).

**Table 1:** Percentages of CD4^+^ and CD8^+^ T cells and their subsets in the TDLNs of HNSCC patients

Cell subset	Min	Max	Median	Mean ± SD
CD4^+^	24.8	75.4	45.5	46.3 ± 10.5
CD4T cell subsets				
CD4^+^ TNF-α^+^	7.9	86.1	63.6	57.3 ± 16.3
CD4^+^TNF-α^hi^	2.7	54.6	38.3	35.7 ± 13.2
CD4^+^TNF-α^low^	5.2	39.8	21.6	21.2 ± 6.4
CD4^+^IFN-γ^+^	0.6	14.2	3.2	3.8 ± 2.7
CD4^+^IFN-γ^+^TNF-α^+^	0.4	14	3.2	3.6 ± 2.6
CD4^+^IFN-γ^+^TNF-α^−^	0.0	0.8	0.1	0.18 ± 0.17
CD4^+^IL-10^+^	0.2	4.5	0.7	0.9 ± 0.7
CD4^+^IL-17^+^	0.4	4.1	1.3	1.4 ± 0.9
CD4^+^IL-4^+^	0.4	5.8	2.2	2.3 ± 1.3
CD4^+^TGF-β^+^	0.3	50.6	28.6	27.4 ± 11.2
CD4^+^CD25^+^FOXP3^+^	2.7	14.6	5.6	6.4 ± 2.7
CD4^+^CD25^+^FOXP3^+^CD127^low^	1.2	12.3	4.6	5.3 ± 2.5
CD4^+^CD25^−^FOXP3^+^	0.7	9.6	1.4	2.2 ± 1.8
CD4^+^CD25^−^FOXP3^+^CD127^low^	0.5	7.2	1	1.5 ± 1.2
CD8^+^	2.6	16.7	8.5	8.6 ± 3.7
CD8^+^ T cell subsets				
CD8^+^ TNF-α^+^	6.9	81.9	43.7	42.4 ± 17.2
CD8^+^IFN-γ^+^	2.1	64.4	20.5	23.6 ± 14.8
CD8^+^IL-10^+^	0.1	2.1	0.7	0.8 ± 0.5
CD8^+^IL-17^+^	0.1	6.2	0.6	1.0 ± 1.2
CD8^+^IL-4^+^	0.3	15.2	2.5	3.4 ± 3.1
CD8^+^TGF-β^+^	4.6	38	18.7	18.8 ± 8.1

TDLN, tumor draining lymph node; SD, standard deviation; hi, high; TNF-α, tumor necrosis factor alpha; IFN-γ, interferon gamma; TGF-β, transforming growth factor beta

### Association of T cell subpopulations with LN involvement

**Fig. 3:**
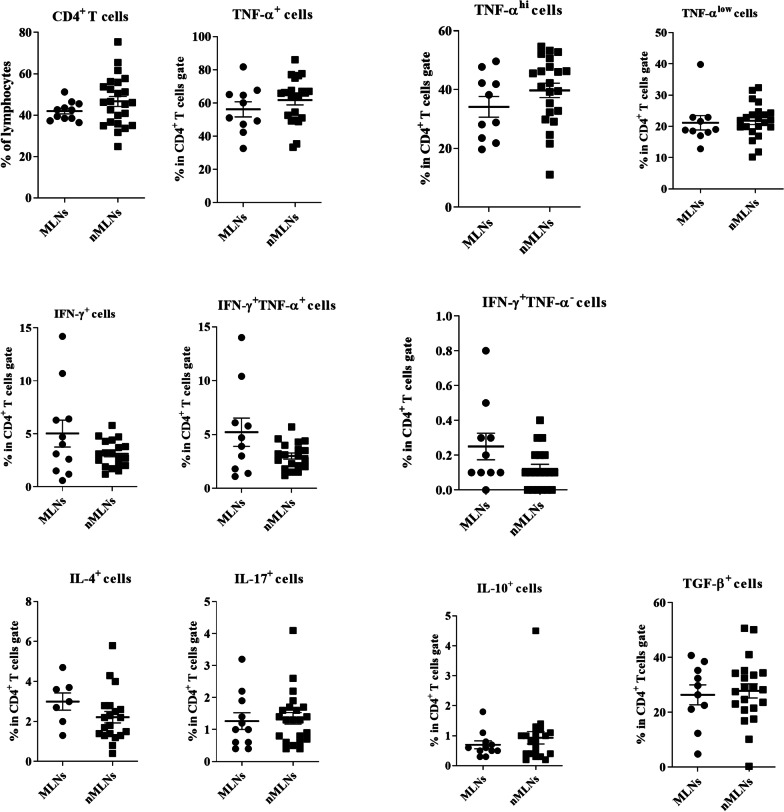
Comparison of the frequencies of CD4^+^ and its subsets in metastatic (MLNs) and non-metastatic lymph nodes (nMLNs) of patients with HNSCC. CD4^+^ T cells gated on the lymphocyte population and then the frequencies of T cell subpopulations were determined within CD4^+^ T cell gate. Horizontal bar is representative of the Mean ± SEM

**Fig. 4:**
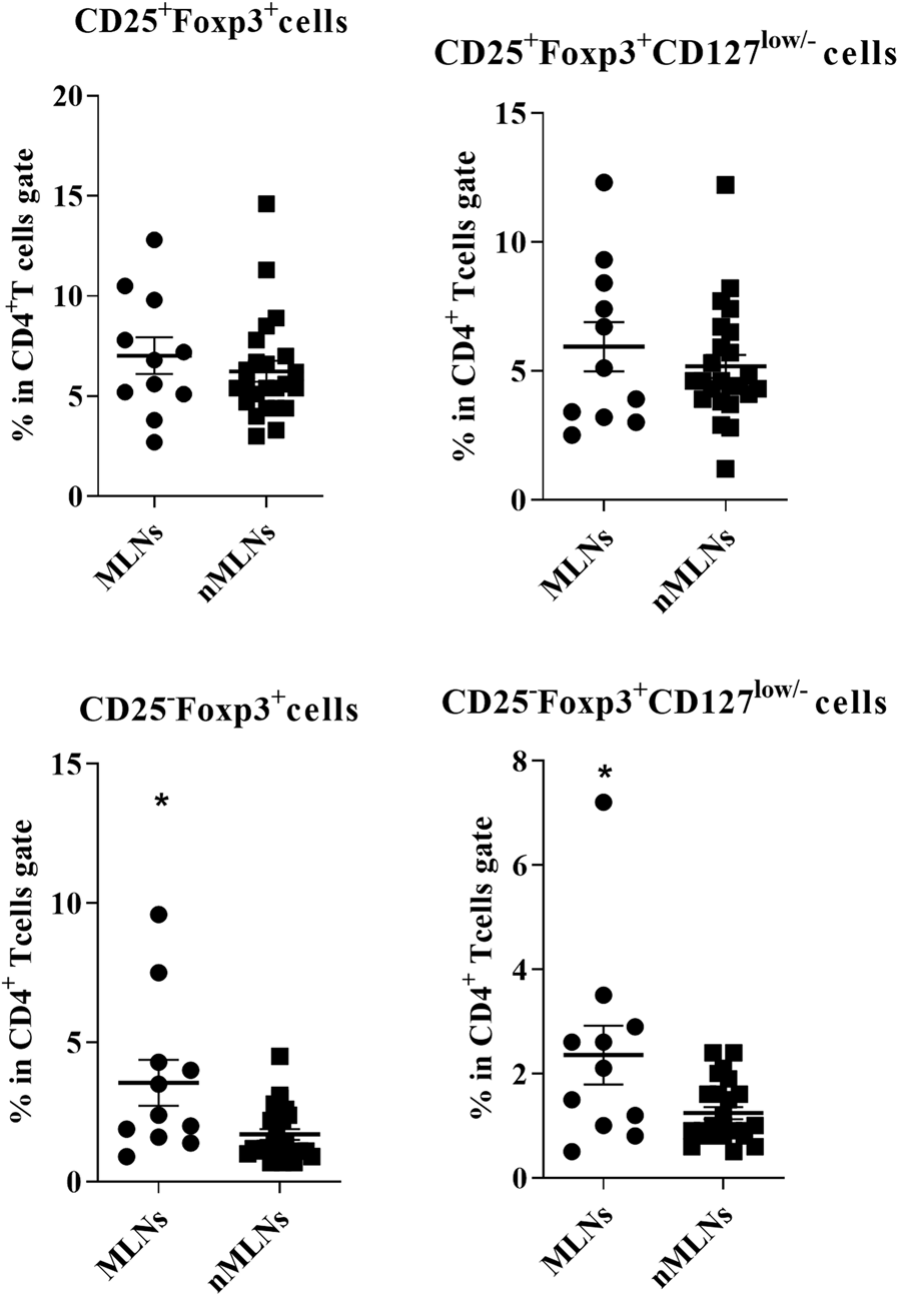
Comparison of the frequencies of CD4^+^ Foxp3^+^ subsets in metastatic (MLNs) and non-metastatic lymph nodes (nMLNs) of patients with HNSCC. CD4^+^ T cells gated on the lymphocyte population and then frequencies of Foxp3^+^ T subsets were determined within CD4^+^ T cell gate. Horizontal bar is representative of the Mean ± SEM. *P value < 0.05

In patients with HNSCC, the frequencies of cytokine-producing CD4^+^ T cells subsets did not show significant changes in metastatic lymph nodes (MLNs) in comparison with non-metastatic LNs (nMLNs) (Fig. 3). Significantly higher percentages of CD4^+^CD25^−^Foxp3^+^ and CD4^+^CD25^−^Foxp3^+^ CD127^low/−^ cells were observed in MLNs (*P* = 0.019 and *P* = 0.040, respectively, Fig. 4). Moreover, there was a direct correlation between the frequency of CD4^+^IFN-γ^+^TNF-α^−^ T cells and the number of involved LNs (R = 0.4, *P* = 0.044).

Further analysis revealed that, in patients with SCC of the tongue, the frequencies of CD4^+^, CD4^+^TNF-α^+^, CD4^+^TNF-α^hi^ and CD4^+^TNF-α^low^ T cells were significantly higher in nMLNs than in MLNs (*P* = 0.015, *P* = 0.019, *P* = 0.017 and *P* = 0.029, respectively, Additional file 1: Fig. S3). In addition, in patients with tongue SCC, the frequency of CD4^+^CD25^+^Foxp3^+^CD127^low/−^ cells was significantly lower in TDLNs of LN^+^ patients (patients with at least one involved LN) in comparison with LN– ones (patients without involved LN, *P* = 0.036, Additional file 1: Fig. S4).

Comparison of the frequencies of different cytokine-producing CD8^+^ T cells and the gMFIs of these cytokines in MLNs and nMLNs of patients with HNSCC, revealed no significant changes. However, significantly higher percentage of IL-10-producing CD8^+^ T cells was observed in MLNs of tongue SCC in comparison with nMLNs (*P* = 0.042, Additional file 1: Fig. S5).

### Comparison of T cell subsets in TDLNs of HNSCC patients according to cancer stage

**Fig. 5:**
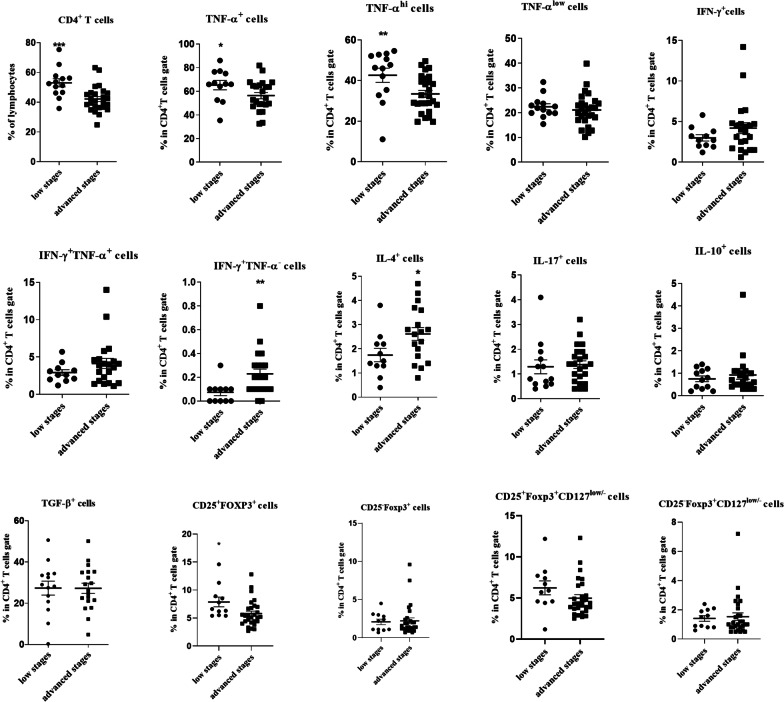
Comparison of CD4^+^ T cells and its subsets in TDLNs of HNSCC patients in low stages (I + II) and advanced stages (III + IV) of the disease CD4^+^ T cells gated on the lymphocyte population and then the frequencies of T cell subpopulations were determined within CD4^+^ T cell gate. Horizontal bar is representative of the Mean ± SEM, *P value < 0.05, **P value < 0.01, ***P value < 0.001

All patients were categorized into two groups: patients in advanced stages (III + IV) and those in low stages (I + II) of the disease. The frequencies of CD4^+^, CD4^+^TNF-α^+^, CD4^+^TNF-α^hi^ T cells were significantly lower in the TDLNs of HNSCC patients in advanced stages (*P* = 0.001, *P* = 0.032 and *P* = 0.010, respectively). In contrast, the frequencies of CD4^+^IFN-γ^+^TNF-α^−^ T cells and CD4^+^IL-4^+^ T cells were significantly higher in advanced stages compared to lower stages of the disease (*P* = 0.005 and *P* = 0.042, respectively, Fig. 5). The percentage of CD4^+^CD25^+^Foxp3^+^ cells showed a significant decrease in advanced stages (*P* = 0.015), similarly, the frequency of CD4^+^CD25^+^Foxp3^+^CD127^low/−^ showed a trend toward decrease in advanced stages of HNSCC (*P* = 0.059, Fig. 5).

**Fig. 6:**
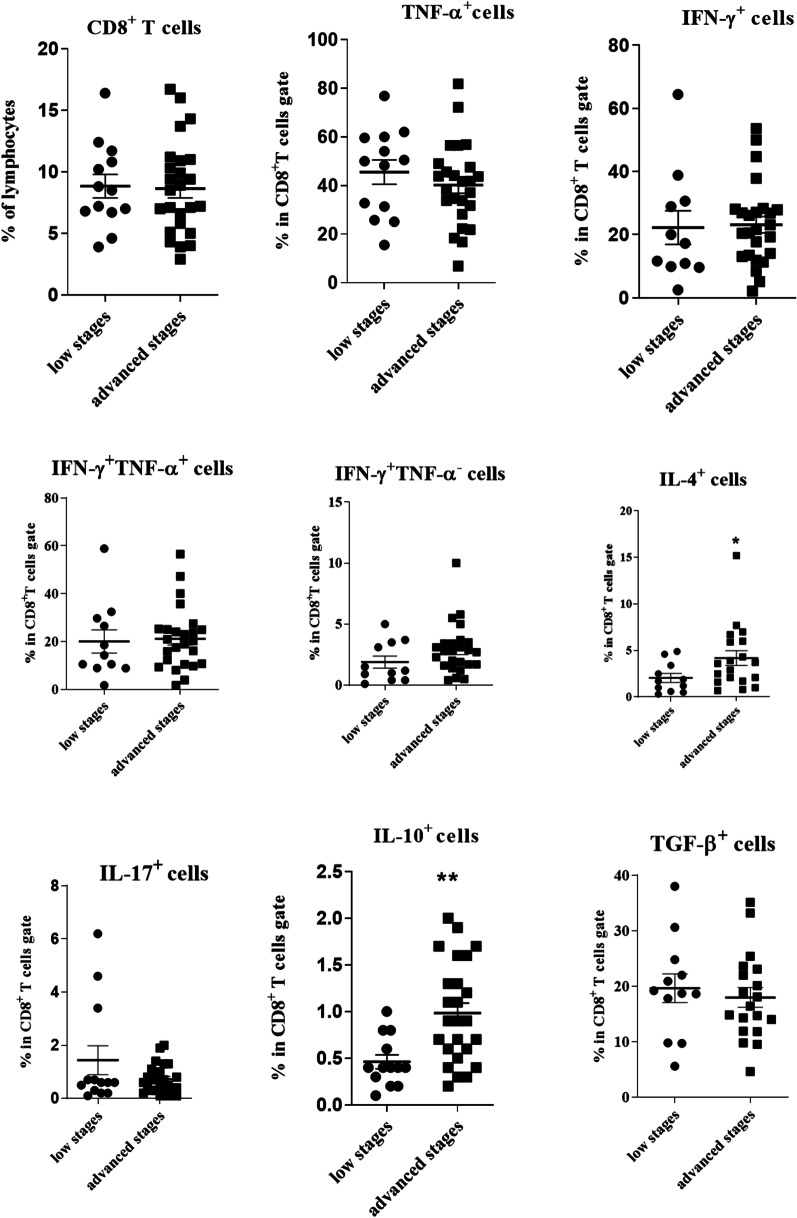
Comparison of CD8^+^ T cells and its subsets in TDLNs of HNSCC patients in low stages (I + II) and advanced stages (III + IV) of the disease. CD8^+^ T cells gated on the lymphocyte population and then the frequencies of T cell subpopulations were determined within CD8^+^ T cell gate. Horizontal bar is representative of the Mean ± SEM, *P value < 0.05, **P value < 0.01

Furthermore, our data showed that the percentages of CD8^+^IL-10^+^ and CD8^+^IL-4^+^ T cells were significantly higher in advanced compared to lower stages in patients with HNSCC (*P* = 0.003 and *P* = 0.043, respectively, Fig. 6).

### Relationships between T cell subsets and tumor grade or size

As only 3 patients with HNSCC were in grade III of the tumor, the frequencies of CD4^+^ T cell subsets were compared in patients with grade I with those with grade II + III of the tumors. Analysis revealed an increasing trend in the percentage of CD4^+^IFN-γ^+^TNF-α^−^ T cells in patients with HNSCC with grade II + III (*P* = 0.079). But the percentages of CD8^+^IFN-γ^+^ and CD8^+^IFN-γ^+^ TNF-α^+^ T cells were significantly lower in grade II + III in comparison with grade I of the tongue SCC (*P* = 0.035 and *P* = 0.043, respectively, Additional file 1: Fig. S6).

**Fig. 7:**
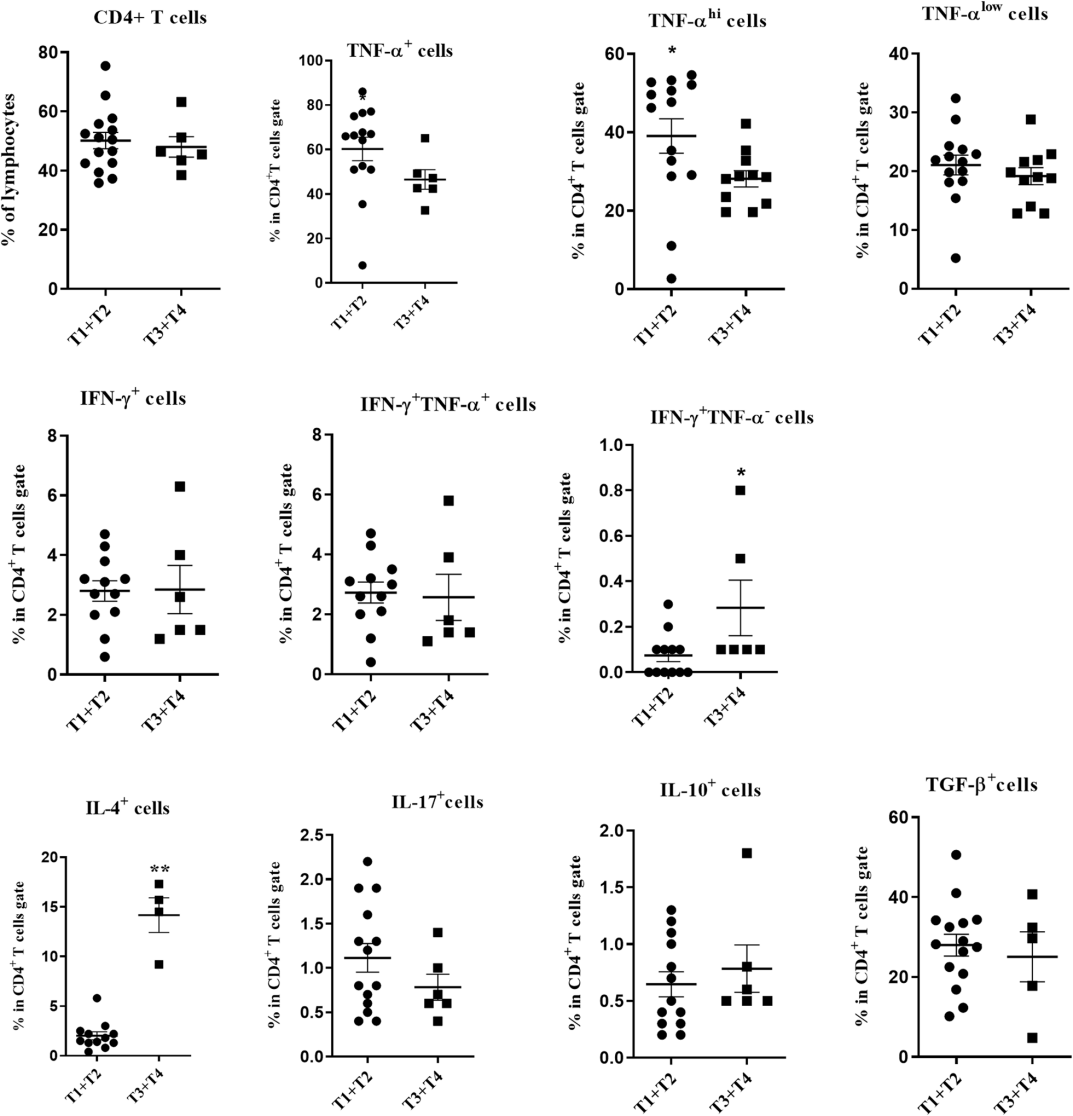
Comparison of CD4^+^ T cells and its subsets in TDLNs of patients with tongue SCC in T1 + T2 and T3 + T4 groups. CD4^+^ T cells gated on the lymphocyte population and then the frequencies of T cell subpopulations were determined within CD4^+^ T cell gate. Horizontal bar is representative of the Mean ± SEM, *P value < 0.05

All patients were categorized into two groups (T1 + T2 and T3 + T4 groups) according to T status of the patients which are based on the size and/or location of the primary tumor. Due to inadequate samples from patients with laryngeal carcinoma, frequencies of different cytokine-producing T cells were only compared in TDLNs of patients with tongue SCC. The percentages of CD4^+^IL-4^+^ and CD4^+^IFN-γ^+^TNF-α^−^ T cells were significantly higher in T3 + T4 than in T1 + T2 group (*P* = 0.001 and *P* = 0.046, respectively). On the other hand, CD4^+^TNF-α^+^, CD4^+^TNF-α^hi^ T cells were significantly lower in patients with T3 + T4 (*P* = 0.026 and *P* = 0.032, respectively, Fig. 7). A significant positive correlation was found between the percentage of CD4^+^IFN-γ^+^TNF-α^−^ T cell and the tumor size (R = 0.4, *P* = 0.020), whereas the frequency of CD4^+^TNF-α^hi^ T cells correlated inversely with the tumor size (R = − 0.3, *P* = 0.045).

**Fig. 8:**
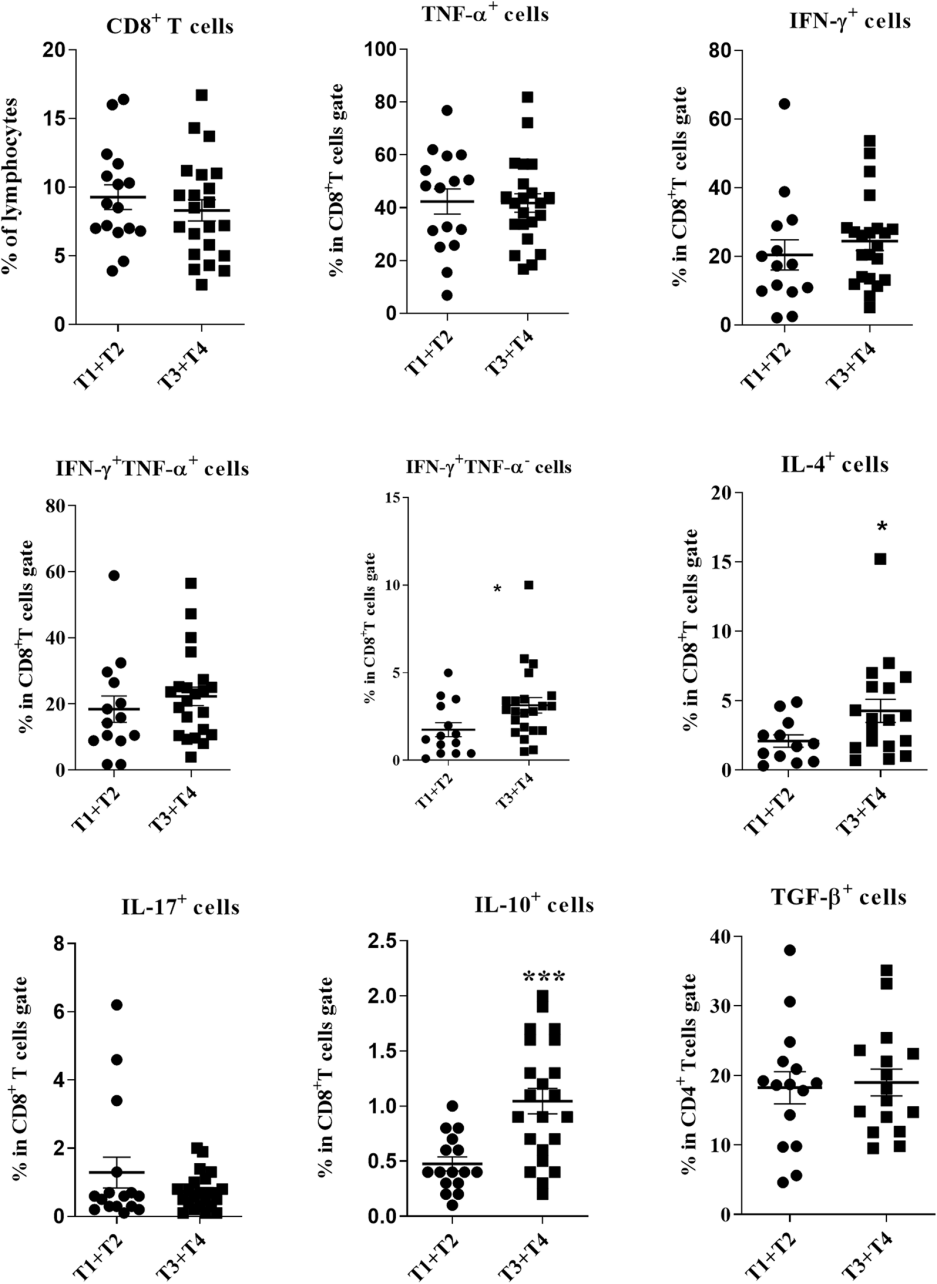
Comparison of CD8^+^ T cells and its subsets in patients with HNSCC in T1 + T2 and T3 + T4 groups. CD8^+^ T cells gated on the lymphocyte population and then the frequencies of T cell subpopulations were determined within CD8^+^ T cell gate. Horizontal bar is representative of the Mean ± SEM, *P value < 0.05. **P value < 0.01. ***P value < 0.001

Assessment of the association of CD8^+^ T cell subsets with tumor size revealed significantly higher percentages of CD8^+^IFN-γ^+^TNF-α^−^, CD8^+^IL-10^+^ and CD8^+^IL-4^+^ T cells in TDLNs of patients with tongue SCC categorized as T3 + T4 compared to T1 + T2 group (*P* = 0.025, *P* = 0.0009 and *P* = 0.046, respectively, Fig. 8). Moreover, our results demonstrated that the frequencies of IL-10^+^CD8^+^ and IL-4^+^CD8^+^ T cells had positive correlations with the tumor size, however the latter was not statistically significant (R = 0.4, *P* = 0.013, R = 0.3, *P* = 0.098, respectively).

### Association of CD4^+^ and CD8^+^ T cells and their subsets with perineural or lympho-vascular invasion

**Fig. 9:**
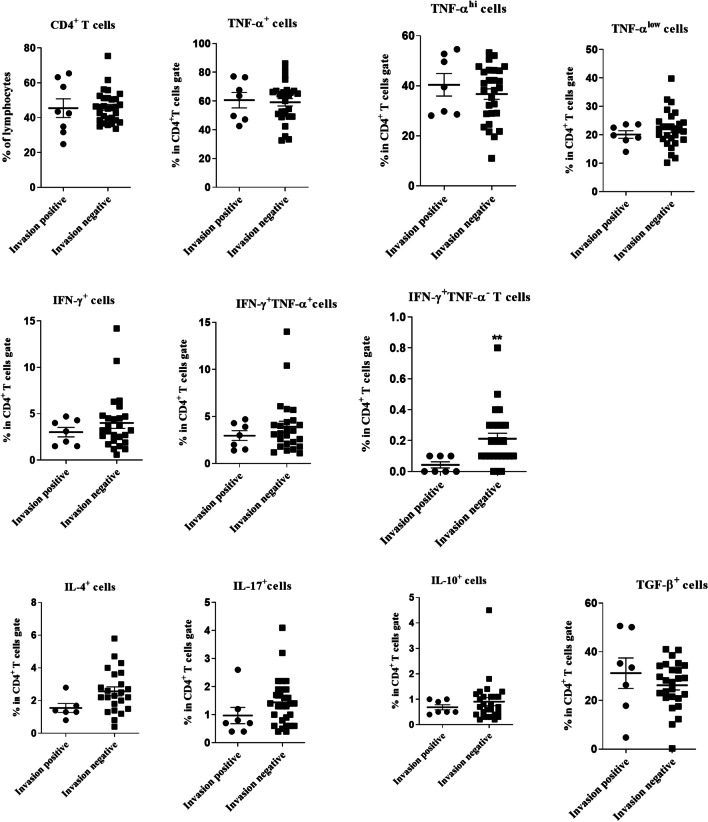
Comparison of CD4^+^ T cells and its subsets in TDLNs of HNSCC patients with or without perineural and/or lymphovascular invasion. CD4^+^ T cells gated on the lymphocyte population and then the frequencies of T cell subpopulations were determined within CD4^+^ T cell gate. Horizontal bar is representative of the Mean ± SEM. *P value < 0.05. **P value < 0.01

A significantly higher percentage of CD4^+^IFN-γ^+^TNF-α^−^ T cells was found in TDLNs of HNSCC patients without lymphatic/vascular invasion (*P* = 0.004). The percentage of CD4^+^IL-4^+^T cells showed a similar but nonsignificant trend (*P* = 0.052, Fig. 9). In tongue SCC, the gMFI of IFN-γ production in CD4^+^T cells was significantly higher in TDLNs of patients with lymphatic/vascular invasion (*P* = 0.015, Additional file 1: Fig. S7). Similar sets of analysis for CD8^+^ T cells showed no significant association between the frequencies of these cells and perineural and/or lymphovascular invasion (Data not shown).

### CD4^+^ and CD8^+^ T cell subpopulations in TDLNs of patients with tongue SCC compared with laryngeal SCC

The percentage of CD4^+^ T cells as well as different CD4^+^ T cell subsets in TDLNs of laryngeal carcinoma were compared with those in TDLNs of tongue SCC. To decrease the effects of confounding factors such as tumor size, LN involvement or disease stage, the percentages were compared in the same stage in the two groups. Due to small sample size such analysis was not feasible in stage I to III. But, in stage IV, the percentage of CD4^+^ T cells was significantly higher in TDLNs of patients with tongue SCC compared to those with laryngeal SCC (*P* = 0.025). In addition, the frequency of CD4^+^IFN-γ^+^TNF-α^+^ T cells showed a non-significant increasing trend in TDLNs of laryngeal compared with tongue SCC (*P* = 0.086). The percentage of CD8^+^TGF-β^+^ T cells was significantly higher in TDLNs of patients with laryngeal SCC than in those with tongue SCC (*P* = 0.018, Additional file 1: Fig. S8).

In addition, the percentages of CD4^+^ T cell subsets were compared in patients with laryngeal and tongue SCCs without LN involvement. CD4^+^CD25^+^Foxp3^+^ and CD4^+^CD25^+^Foxp3^+^CD127^low/−^ were significantly higher in TDLNs of patients with tongue compared to laryngeal SCC (*P* = 0.020 and *P* = 0.025, respectively, Additional file 1: Fig. S9). In contrast, the frequencies of CD4^+^IFN-γ^+^TNF-α^+^, CD4^+^IL-10^+^ and CD4^+^IL-17^+^ cells exhibited increasing trends in TDLNs of patients with laryngeal SCC (*P* = 0.098, *P* = 0.075 and *P* = 0.068, respectively). The percentage of IL-10- producing CD8^+^ T cells was significantly higher in TDLNs of patients with laryngeal compared to tongue SCC (*P* = 0.007, Additional file 1: Fig. S10). Similar results were obtained in patients with laryngeal and tongue SCCs with tumor size ≤ 3 cm. The frequencies of CD4^+^IL-4^+^, CD4^+^IL-17^+^ and CD8^+^IL-10^+^ showed significant increases in laryngeal SCC patients with tumor size ≤ 3 cm (*P* = 0.024, *P* = 0.048, *P* = 0.001, respectively) (Additional file 1: Fig. S11 and S12). However, CD4^+^CD25^+^Foxp3^+^ and CD4^+^CD25^+^Foxp3^+^CD127^low/−^ were significantly higher in TDLNs of patients with tongue compared to laryngeal SCC (*P* = 0.013, *P* = 0.031) (Additional file 1: Fig. S13).

The associations between the frequencies of T cell subsets and clinicopathological parameters of HNSCC were summarized in Table 2.

**Table 2:** Association of frequencies of T cell subsets in TDLNs of HNSCC cancer patients with prognostic indicators

T cell subsets	Association with clinic-pathological characteristics
CD4^+^ T	Higher frequency in nMLNs of tongue SCC, higher frequency in low stages
CD4^+^TNF-α^+^	Higher frequency in nMLNs of tongue SCC, higher frequency in low stages, higher frequency in tongue cancer patients with smaller tumor size
CD4^+^TNF-α^hi^	higher frequency in nMLNs of tongue SCC, higher frequency in low stages, higher frequency in tongue cancer patients with smaller tumor size, negative correlation with tumor size
CD4^+^TNF-α^low^	Higher frequency in nMLNs of tongue SCC
CD4^+^IFN-γ^+^	Higher gMFI of IFN-γ in tongue cancer patients with lymphatic/vascular invasion
CD4^+^IFN-γ^+^TNF-α^−^	Positive correlations with the number of involved LNs and tumor size higher frequency in advanced stages and in patients without tumor lymphatic/vascular invasion
CD4^+^IL-4^+^	Higher frequency in advanced stages and in tongue cancer patients with larger tumor size
CD4^+^TGF-β^+^	Higher gMFI of TGF-β in tongue cancer patients with larger tumor size
CD4^+^CD25^+^Foxp3^+^	Higher frequency in low stages
CD4^+^CD25^+^Foxp3^+^CD127^low/−^	Higher frequency in LN‒ patients with tongue SCC
CD4^+^CD25^−^Foxp3^+^	Higher frequency in MLNs
CD4^+^CD25^−^Foxp3^+^ CD127^low/−^	Higher frequency in MLNs
CD8^+^IFN-γ^+^	Higher frequency in low grade
CD8^+^IFN-γ^+^ TNF-α^+^	Higher frequency in low grade
CD8^+^IFN-γ^+^TNF-α^−^	Higher frequency in patients with larger tumor size
CD8^+^IL-4^+^	Higher frequency in advanced stages and in patients with lager tumor size
CD8^+^IL-17^+^	High gMFI of IL-17 in patients at stage III compared to stageIV
CD8^+^IL-10^+^	Higher frequency in MLNs of tongue SCC, higher frequency in advanced stages and in patients with larger tumor size, positive correlation with tumor size

## Discussion

Our previous studies have shown changes in immune profile of the TDLNs upon disease progression and LN involvement in breast, bladder or head and neck cancers [28–31]. Here, we assessed the frequencies of different cytokine-producing CD4^+^ and CD8^+^ T cell subpopulations in TDLNs of tongue and laryngeal SCC. We sought to determine how the tumor progression and invasion to adjacent draining LNs can affect T cell subpopulations in these LNs.

In this study, CD4^+^TNF-α^+^ T cells showed association with markers of a good prognosis, as its frequency was found to be higher in early HNSCC stages and in nMLNs. In contrary, IL-4-producing CD4^+^ T cells was higher in advanced stages and in tongue SCC patients with larger tumor size which are linked to a poor prognosis. These results suggest that Th1 cells that secret TNF-α may be predominant in patients without lymph node involvement and those with low disease stages, whereas patients with LN metastasis and with advanced stages have an increased Th2 response. However, caution must be taken when comparing patients in higher stages and those in lower stages or patients with and without LN involvement, as confounding factors such as age or sex can interfere and complicate the interpretation. In fact, by following patients, one can get better understanding of this complex relationship over time and it helps to infer how the nature of the immune response evolves during tumor progression, but such longitudinal studies are almost impossible in human because once disease is diagnosed the patient undergoes surgery and/or receives medication.

In agreement with our results, several studies in human and mouse HNSCC have reported that with cancer progression, immune responses tend to shift toward a Th2 phenotype [11–13]. In a high-throughput gene expression assay of HNSCC tumor, increased expression of IL-4 has been reported [32]. A decrease in the level of CD4^+^ T cells has been shown to be gone along with a shift to the Th2 phenotype (high level of IL-4 and low level of IL-2) in peripheral blood mononuclear cells of patients with HNSCC when compared with normal controls [33]. Collectively, these data provide evidence for a tumor-mediated modulation of immune responses in patients with HNSCC. These data further support the “Th2-skewing hypothesis of tumor escape”. It has been known since the 1950s that cellular and/or humoral immune response are dependent on the dose of antigen [34], a finding supported by many investigations, including cancer studies [35, 36]. In 1980s, North showed that in mouse model of fibrosarcoma a lethal dose of tumor antigen initially produces anti-tumor cytotoxic T lymphocytes (CTL) response which is responsible for “concomitant immunity” and CTL response is inhibited during tumor progression by suppressor T cells [35, 36]. In the 1960s, it was shown in mouse tumor models that induction of cellular immunity was linked to immune resistance in tumor and production of IgG promoted tumor growth [37]. Since Th2 cell-mediated immune responses are involved in IgG antibody production, these older data appear to be relevant, especially as the role of cancer cell-derived IgG response in tumor progression has been recently reported in various cancers [38].

TNF-α is a cytokine that affects the function of different cell types and produced by immune and non-immune cells. It has been initially described as a pro-inflammatory cytokine; however, it has become evident that this cytokine modulates multiple biological processes from immune responses to cell survival, differentiation and mitochondrial function [39, 40]. The functional outcome of TNF-α signaling depends on various factors including its dose and duration of exposure, soluble and membrane-bound form, receptor binding (TNFR1 and TNFR2) and co-stimulation with other cytokines or mediators [41]. Yet, the role of TNF-α in cancer progression is still a matter of debate. It has been shown that low-dose chronic TNF-α production is a feature of many cancers during which TNF-α promotes cancer growth, invasion, and metastasis through different mechanisms [42, 43]. Several studies have shown that chronic low-level TNF-α can promote angiogenesis, whereas high doses of TNF-α act as antiangiogenic agent [44–46]. In this study, we found significant inverse associations between the percentage of TNF-αhi T cells and LN metastasis, stage of the disease and tumor size which are all markers of a poor prognosis. While, the frequency of CD4^+^IFN-γ^+^TNF-α^−^ T cells was related to poor prognostic markers as it was higher in patients with larger tumor size and higher stage and showed direct correlation with the number of involved LNs. In a study on breast cancer, the frequency of TNF-αhi B cells was negatively linked to the number of involved LNs [31]. As it was mentioned, TNF-α at high dose can play a role against malignant growth. It was found that high level of TNF can inhibit tumor cell growth via inhibition of vascularization, direct cytotoxicity and promoting anti-tumor immunity [43, 47, 48].

A study demonstrated that IFN-γ downregulates the expression of CXCR4 in HNSCC cell lines and consequently inhibits tumor cell migration and proliferation [49]. In addition, it has been reported that higher serum levels of IFN‐γ in HNSCC patients were associated with good prognostic factors. Higher IFN‐γ expression was found in patients in early stages and without LN involvement [50]. However, results of the present study showed that higher intensities of IFN-γ in CD4^+^ lymphocytes showed associations with perineural/lymphovascular and also LN invasion. Although no significant correlation was found between the frequency of CD4^+^IFN-γ^+^TNF-α^+^ T cells and disease parameters, the presence of IFN-γ monofunctional T cells (CD4^+^IFN-γ^+^TNF-α^−^ T cells) in TDLNs of HNSCC had the most associations with poor prognostic indicators.

Polyfunctional Th1 cells simultaneously produce two or more cytokines including IFN-γ, IL-2, and TNF-α. Recently, it has been shown that multifunctional T cells are associated with more efficient function than monofunctional T cells (T cells produce only one cytokine) [51]. Polyfunctional Th1 cells have been associated with favorable disease outcome in a number of infectious diseases and cancers. These cells have been strongly linked to higher protective efficacy after vaccination compared with IFN-γ-producing monofunctional T cells [52–56].

The positive association of IFN-γ expression and disease-free survival has been reported in patients with various cancers [57–59]. However, studies have shown that tumor cells can escape the immune system by not responding to IFN-γ signals, which helps tumor cells block the anti-proliferative and immunomodulatory effect of IFN-γ [60, 61]. IFN-γ can promote tumor growth by downregulation of MHC molecules, promoting angiogenesis and eliciting the expression of tolerant molecules such as IDO and checkpoint inhibitors like PD-L1 and TIM3 [62]. In the tumor microenvironment of HNSCC approximately 50–60% of tumors express PD‐L1 which is stimulated by the increase of IFN‐γ in the tumor microenvironment (TME) [63]. Its role also depends on cellular sources and presence of other cytokines and factors. In line with this conclusion, in this study, we found that the percentage of IFN-γ^+^TNF-α^−^ T cells mostly related to poor prognostic factors such as LN involvement, advanced stage of the disease and size of the tumor while the frequency of IFN-γ^+^TNF-α^+^ T cells showed no associations with the indicators of unfavorable prognosis. Consequently, IFN-γ can have complex, multifaceted roles in the context of different tumors that needs further investigations.

Another finding of this study was the association between the frequencies of regulatory T cell subset in TDLNs of HNSCC patients and prognostic indicators. We found significant direct correlations between the percentages of CD4^+^CD25^+^Foxp3^+^ and CD4^+^CD25^+^Foxp3^+^CD127^low/−^ cells and lower stages of the disease and absence of LN involvement. Several studies have focused on the role of Foxp3^+^Tregs in HNSCC but their results were controversial. A number of studies reported that CD4^+^Foxp3^+^ regulatory T cell frequency was significantly higher in peripheral blood and tumor tissues of patients with HNSCC than in healthy controls and it was correlated with progression of HNSCC [64–67]. However, in some other studies Tregs showed no association with prognosis [68, 69] or even correlated with improved outcome [70–72]. In a study of patients with squamous cell carcinoma of the oro- and hypopharynx no association was found between patients’ outcome and the frequency of Foxp3^+^Tregs in any compartment of the tumor [68]. In contrast, Bron et al. reported increased intratumoral Foxp3^+^CD4^+^ T cells compared to peripheral blood lymphocytes from HNSCC patients and healthy donors. They reported a positive correlation between the higher levels of tumor-infiltrating Tregs and the absence of locoregional metastases [70]. Recently, it has been reported that the suppressive activity of peripheral CD25int/hiCD127low/− Tregs increased in patients with laryngeal and oropharyngeal carcinoma and was associated with advanced stage and nodal involvement [73].

The activity of Treg cells may be affected by the type of cancer. In colon, bladder or head and neck cancers with the phenotype of chronic inflammation, infiltration of Tregs within tumor was described to be associated with prognosis and favorable outcome, [74–76] likely through the down-regulation of tumor-promoting inflammatory reactions [74]. In the present study, higher numbers of CD25^+^Foxp3^+^ and CD25^+^Foxp3^+^CD127^low/−^ regulatory T cells in TDLNs of HNSCC correlated with good prognostic factors. In our previous study, different B cell subsets with regulatory phenotype showed association with good prognosticators of HNSCC [28]. These results might be partially attributed to the capacity of regulatory cells to down-regulate the inflammatory responses, which are responsible for the tumor progression.

CD4^+^CD25^−^Foxp3^+^ T cell subsets were first described in peripheral blood of patients with autoimmune diseases [77–79]. They are either considered dysfunctional Treg cells, or activated effector T cells with transient expression of Foxp3 [77, 80]. It has been suggested that CD4^+^CD25^−^FoxP3^+^ cells may be a heterogeneous population with effector or suppressive phenotype [81].

Numerous studies have reported the presence of CD4^+^CD25^+^Foxp3^+^ Treg cells in HNSCC [64–66, 68–70, 74, 82, 83]. However, there are limited data about CD4^+^CD25^−^Foxp3^+^ Treg cells in HNSCC. A study by Bergmann et al. in TILs and PBMC of HNSCC patients demonstrated a significant increase in the frequency and inhibitory activity of CD4^+^CD25^−^Foxp3^+^ T cells in patients presenting with advanced stages and it was suggested that the tumor microenvironment may have a role in the induction of CD4^+^CD25^−^Foxp3^+^ T cells within tumor [84]. In the present study, the presence of CD4^+^CD25^−^Foxp3^+^ T cell subset was shown in TDLNs of patients with HNSCC. However, their frequency was significantly lower than CD4^+^CD25^+^FoxP3^+^ cells. Intensity of Foxp3 expression was significantly lower in the CD25^−^Foxp3^+^ subset compared to CD25^+^Foxp3^+^ cells. Similar results were also reported in breast and colon cancer [81, 85]. In addition, we found that the frequencies of CD4^+^CD25^−^Foxp3^+^ and CD4^+^CD25^−^Foxp3^+^CD127^low/−^ cells were significantly higher in MLNs of HNSCC patients as compared with nMLNs. However, whether these cells have a suppressive function in HNSCC requires more investigations.

In HNSCC, cytotoxic CD8^+^ T cells infiltrates have been most often associated with favorable prognosis [69, 86, 87]. However, PD-1 has been reported to have a higher expression on tumor-infiltrating CD8^+^ T cells in HNSCC, which resulted in dysfunction of PD-1^+^CD8^+^ T cells and tumor growth progression [88].

The percentages of IL-17- and IL-4-producing CD8^+^ T cells showed positive association with breast cancer progression and also down-regulation of IFN-γ in these cells was associated with metastasis to the draining LNs [89]. In bladder cancer, the presence of double positive IFN-γ/IL-17-producing CD8^+^ T cells was reported to directly correlate with LN involvement. A positive association was also found between the frequency of IFN-γ-producing CD8^+^ T cells and high histological grade [30]. However, in the present study, significant reverse correlation was found between the frequencies of CD8^+^IFN-γ^+^ (monofunctional T cells) and CD8^+^IFN-γ^+^TNF-α^+^ T cells (polyfunctional T cells) and higher cancer stage. On the other hand, it was demonstrated that along with the metastasis of the tumor cells into the draining LNs and progression of the disease to advanced stages, the frequencies of CD8^+^ T cells expressing Tc2-associated cytokines (IL-4, IL-10) increased. The two aforementioned subsets and also CD8^+^IFN-γ^+^TNF-α^−^ cells correlated directly with the tumor size -another poor prognosticator. These findings may suggest a negative role for cells with Tc2-related phenotype in HNSCC.

## Conclusion

The present study evaluated the immune profile of TDLNs of HNSCC during disease progression. Collectively among T cell subsets, CD4^+^IFN-γ^+^TNF-α^−^, CD4^+^TNF-α^+^, CD4^+^IL-4^+^ and CD8^+^IL-4^+^, CD8^+^IL-10^+^ T cells showed the most associations with disease parameters. The frequencies of CD4^+^IFN-γ^+^TNF-α^−^ and CD8^+^IL-10^+^ T cells showed the most positive associations with the parameters known to be indicators of a poor prognosis such as LNs involvement, larger tumor size and higher stage of the disease. Moreover, CD8^+^IL-4^+^ and CD4^+^IL-4^+^ T cells had positive associations with tumor size and stage of the disease. In contrast, CD4^+^TNF-α^+^ T cells showed the most positive associations with the indicators of a more favorable prognosis. On the other hand, Foxp3^+^ Treg cells were found to be associated with good prognostic indicators. Collectively, the findings of this study provided more evidence for the significance of the immune profile of TDLNs in HNSCC for determining cancer prognosis; however further investigations with larger sample size are required to establish the immune profile of HNSCC as an independent prognostic factor. In addition, functional studies using sorted immune cells and investigation of the frequencies and function of the innate immune cells such as DC and NK cells in TDLNs of the HNSCC are warranted to give us a more comprehensive picture of the immune reactions in the TDLNs. Another issue which is worth noting is that HPV status of HNSCC patients could influence their immune response, however, we could not assess HPV infection in our patients. This can be addressed as a limitation to this study and a potential aspect for further investigation.

## Material and methods

### Subjects

Cervical lymph node (LN) samples were taken from 39 patients with squamous cell carcinomas of tongue and larynx (Clinical and pathological characteristics of the patients were summarized in Table 3). Patients who did not receive chemo or radiotherapy were enrolled in this study after signing an informed consents in written. All procedures performed in the current study were approved by ethics committee of Shiraz University of Medical Sciences (IR.SUMS.REC.1396.S664) in accordance with the 1964 Helsinki declaration and its later amendments.

**Table 3:** Clinico-pathological characteristics of HNSCC patients enrolled in the study of T cell profile

Characteristics	Value
Age (years)	61.2 ± 15.8 (27–88)
Gender	
Male	28 (71.8%)
Female	11 (28.2%)
Tumor Type	
Larynx	17 (43.6%)
Tongue (from oral cavity)	22 (56.4%)
Lymph node (LN) Status(greatest dimension, cm)	
N0 (free LNs)	24 (61.5%)
N1 (≤ 3)	4 (10.3%)
N2 (> 3–6)	9 (23.1%)
N3 (> 6)	1 (2.6%)
Unknown	1 (2.6%)
Tumor size	
T1	4 (10.3%)
T2	12 (30.8%)
T3	15 (38.5%)
T4	7 (17.9%)
Unknown	1(2.6%)
Stage	
I	4 (10.3%)
II	9 (23.1%)
III	10 (25.6%)
IV	15 (38.5%)
Unknown	1 (2.6%)
Histological grade*	
Well differentiated (I)	19 (48.7%)
Moderately differentiated (II)	15 (38.5%)
Poorly differentiated (III)	3 (7.7%)
Unknown	2 (5.1%)
Perineural/lymphovascular invasion	
Positive	8 (20.5%)
Negative	30 (76.9%)
Unknown	1 (2.6%)
Lymph nodes characteristic	
MLNs	11 (28.2%)
nMLNs	24 (61.5%)
Not determined	4 (10.3%)

Based on Bryne’s (1989, 1992) (ITF) Invasive Tumor Front Grading System

MLN, metastatic lymph node; nMLN, non-metastatic lymph node

### Flow cytometry analysis of T cell subsets

#### Antibodies

We isolated mononuclear cells from fresh LN samples, as previously described [28]. Phenotypic & cytokine analysis of T cells was performed using FACSCalibur flow cytometer (BD biosciences, USA). The following mouse anti-human monoclonal antibodies (mAbs) were used: PerCp-Cy5.5 conjugated anti-CD4 (Clone: RPA-T4), PerCp-Cy5.5 conjugated anti-CD8 (Clone: SK1), Fluorescein isothiocyanate (FITC)-conjugated anti-CD25 (Clone: M-A251), Allophycocyanin (APC)-conjugated anti-CD127 (Clone: A019D5), FITC-conjugated anti-IFN-γ (Clone: 4S.B3), PE anti-human TGF-β (Clone: TW4-9E7), APC-conjugated anti-IL-17 (Clone: BL168), APC-conjugated anti-IL-4 (Clone: 8D4-8), APC-conjugated anti-IL-10 (Clone: JES3-19F1), and their respective isotype controls were purchased from Biolegend, USA. Phycoerythrin (PE)-conjugated anti-Foxp3 (Clone: 259 D/C7), PE-conjugated anti-tumor necrosis factor-α (TNF-α) (Clone: MAB11) and their isotype controls were obtained from BD biosciences.

#### Lymphocytes activation

For the evaluation of the expression of IL-4, IFN-ɣ, TNF-α and IL-17, by CD8^+^ or CD4^+^ T cells, mononuclear cells were stimulated for 5 h with phorbol 12-myristate 13-acetate (PMA) (50 ng/ml) and Ionomycine (1 μg/ml) (both from Sigma-Alderich, Germany) in the presence of Berefeldin A (1 μl/ml, BD Bioscience). For the assessment of the IL-10 expression, cells were stimulated with PMA (20 ng/ml) and Ionomycin (1 μg/ml) for 5 h in the presence of Berefeldin A (1 μl/ml). Moreover, we stimulated cells with phytohemagglutinin (PHA) (1 μl/ml) (Sigma-Alderich,) for 24 h followed by addition of Berefeldin A for the last 5 h to assess the expression of TGF-β in T cells.

#### Surface and intracellular staining

Stimulated cells were incubated with anti-CD8 antibody. After cells were fixed using 1% paraformaldehyde (Sigma, Germany) and permeabilized using Perm/Wash buffer (Biolegend, USA). The permeabilized cells were then stained with anti-IFN-ɣ, anti-TNF-α, anti-IL-4, anti-TGF-β, anti-IL-10 and anti-IL-17 or their associated isotype controls. For the evaluation of the cytokine expression in CD4^+^ T cells, cells were fixed and permeabilized and stained with anti-CD4 and antibodies for cytokines (mentioned above) or their associated isotype controls.

To assess the frequency of Tregs, isolated mononuclear cells were stained with anti-CD4, anti-CD25 and anti-CD127 antibodies or associated isotype controls, and then fixed and permeabilized with Foxp3 buffer set (BD biosciences) and stained with anti-Foxp3 antibody.

#### Flow cytometry data analysis

Data were acquired on FACSCalibur flow cytometer (BD biosciences). Flow cytometry data were analyzed by FlowJo software (version 7.6.2, Ashland,San Diego CA, USA). First, lymphocytes were gated according to their forward and side scatters. After assessment of the frequency of CD4^+^ or CD8^+^ T cells in the lymphocyte gate, the frequencies of various T cell subgroups were determined as the percentages of CD4^+^ or CD8^+^ lymphocytes expressing each molecule. Geometric Mean Florescence Intensities (gMFI) of cytokines were calculated to assess their per cell production. Data were normalized using the gMFI of the cytokine negative cell population.

### Statistical analysis

Kolmogorov–Smirnov, Shapiro–Wilk and D'Agostino's K-squared tests were performed for the assessment of the normal distribution of data. In each test group, some data sets passed normality test and some did not. In addition, as the sample sizes in different groups (i.e.: metastatic and nonmetastatic LNs, N1, N2 and N3, T1 and T2 …) were relatively small and also were not equal, we preferred to use non-parametric Mann–Whitney U, Kruskal–wallis H and Dunn’s posttest for the comparisons of the quantitative data between two and more than two groups, respectively. Correlation analysis was calculated using spearman’s ranks test. Analysis was done using the SPSS 16 software (version 16, SPSS Inc, USA) and the *P* values < 0.05 were considered significant. Graphs were prepared using GraphPad Prism 6 software (Inc; San Diego CA, USA, 2015).

## Supplementary Information

**Additional file 1: Fig. S1.** Flow cytometry analysis of unstimulated CD4^+^ T cell subsets in the tumor draining lymph nodes of patients with HNSCC. Lymphocytes were gated (A) CD4^+^ T cells were gated followed by defining these subpopulations in CD4^+^ T cells gate: (B) TNF-α^+^ cells (C) IFN-γ^+^ cells (D) IL-4^+^ cells (E) IL-17^+^ cells (F) IL-10^+^ cells (G) TGF-β^+^ cells subsets according to their cognate cytokine expression. **Fig. S2.** Flow cytometry analysis of unstimulated CD8^+^ T cell subsets in the tumor draining lymph nodes of patients with HNSCC. (A) Lymphocytes were gated (B) CD8^+^ T cells were gated followed by defining these subpopulations in CD8^+^ T cells gate: (C) TNF-α^+^ cells (D) IFN-γ^+^ cells (E) IL-4^+^ cells (E) IL-17^+^ cells (F) IL-10^+^ cells (G) TGF-β^+^ cells subsets according to their cognate cytokine expression. **Fig. S3.** Comparison of the frequencies of CD4^+^ and its subsets in metastatic (MLNs) and non-metastatic lymph nodes (nMLNs) of patients with tongue SCC. CD4^+^ T cells gated on the lymphocyte population and then the frequencies of T cell subpopulations were determined within CD4^+^ T cell gate. Horizontal bar is representative of the Mean ± SEM. *P value < 0.05. **Fig. S4.** Comparison of the frequencies of CD4^+^ Foxp3^+^ subsets in patients with tongue SCC with at least one involved lymph node (LN +) or without lymph node involvement (LN‒). CD4^+^ T cells gated on the lymphocyte population and then the frequencies of T cell subpopulations were determined within CD4^+^ T cell gate. Horizontal bar is representative of the Mean ± SEM. *P value < 0.05. **Fig. S5.** Comparison of the frequencies of CD8^+^ and its subsets in metastatic (MLNs) and non-metastatic lymph nodes (nMLNs) of patients with tongue SCC. CD8^+^ T cells gated on the lymphocyte population and then the frequencies of T cell subpopulations were determined within CD8^+^ T cell gate. Horizontal bar is representative of the Mean ± SEM. *P value < 0.05. **Fig. S6.** Comparison of CD8^+^ T cells and its subsets in patients with tongue SCC with tumor grade I and grade II + III. CD8^+^ T cells gated on the lymphocyte population and then the frequencies of T cell subpopulations were determined within CD8^+^ T cell gate. Horizontal bar is representative of the Mean ± SEM, *P value < 0.05. **Fig. S7.** Comparison of gMFI of TNF-α, IFN-γ, IL-4, IL-17, IL-10 and TGF-β in CD4^+^ lymphocytes in TDLNs of tongue SCC with or without perineural and/or lymphovascular invasion. Horizontal bar is representative of the Mean ± SEM. *P value < 0.05. **Fig. S8.** Comparison of CD8^+^ T cells and its subsets in TDLNs of tongue and laryngeal SCC with the stage IV of disease. Horizontal bar is representative of the Mean ± SEM, *P value < 0.05. **Fig. S9.** Comparison of CD4^+^Foxp3^+^T cells subsets in TDLNs of tongue and laryngeal SCC without LN involvement. Horizontal bar is representative of the Mean ± SEM, *P value < 0.05. **Fig. S10.** Comparison of CD4^+^ T cells subsets in TDLNs of tongue and laryngeal SCC without LN involvement. Horizontal bar is representative of the Mean ± SEM, *P value < 0.05, **P value < 0.01. **Fig. S11.** Comparison of CD4^+^ T cells and its subsets in TDLNs of tongue and laryngeal SCC with tumor size ≤ 3 cm. Horizontal bar is representative of the Mean ± SEM, *P value < 0.05. **Fig. S12.** Comparison of CD8^+^ T cells and its subsets in TDLNs of tongue and laryngeal SCC with tumor size ≤ 3 cm. Horizontal bar is representative of the Mean ± SEM, **P value < 0.01. **Fig. S13.** Comparison of CD4^+^Foxp3.^+^T cells subsets in TDLNs of tongue and laryngeal SCC with tumor size ≤ 3 cm. Horizontal bar is representative of the Mean ± SEM, *P value < 0.05

## Data Availability

The datasets generated and/or analyzed during the current study are not publicly available due to privacy and ethical concerns but are available from the corresponding author on reasonable request.

## Abbreviations

HNSCC: Head and neck squamous cell carcinoma
TDLN: Tumor draining lymph node
OSCC: Oral squamous cell carcinoma
Th1: T helper 1
CTLs: Cytotoxic T cells
TNF-α: Tumor necrosis factorα
IFN-γ: Interferon-γ
Tfh: T follicular helper cells
TGF-β: Transforming growth factor β
PMA: Phorbol 12-myristate 13-acetate
PHA: Phytohemagglutinin
gMFI: Geometric mean florescence intensities
MLNs: Metastatic lymph nodes
nMLNs: Non metastatic lymph nodes
TME: Tumor microenvironment
