# Supplementary material for: Regulatory and effector T cell subsets in tumor-draining lymph nodes of patients with squamous cell carcinoma of head and neck
Source: BMC Immunol. 2022 Nov 15;23:56. doi: 10.1186/s12865-022-00530-3 (PMC9664675; doi:10.1186/s12865-022-00530-3)
Supplement: Supplementary file 1 — Additional file 1: Fig. S1. Flow cytometry analysis of unstimulated CD4+ T cell subsets in the tumor draining lymph nodes of patients with HNSCC. Lymphocytes were gated (A) CD4+ T cells were gated followed by defining these subpopulations in CD4+ T cells gate: (B) TNF-α+ cells (C) IFN-γ+ cells (D) IL-4+ cells (E) IL-17+ cells (F) IL-10+ cells (G) TGF-β+ cells subsets according to their cognate cytokine expression. Fig. S2. Flow cytometry analysis of unstimulated CD8+ T cell subsets in the tumor draining lymph nodes of patients with HNSCC. (A) Lymphocytes were gated (B) CD8+ T cells were gated followed by defining these subpopulations in CD8+ T cells gate: (C) TNF-α+ cells (D) IFN-γ+ cells (E) IL-4+ cells (E) IL-17+ cells (F) IL-10+ cells (G) TGF-β+ cells subsets according to their cognate cytokine expression. Fig. S3. Comparison of the frequencies of CD4+ and its subsets in metastatic (MLNs) and non-metastatic lymph nodes (nMLNs) of patients with tongue SCC. CD4+ T cells gated on the lymphocyte population and then the frequencies of T cell subpopulations were determined within CD4+ T cell gate. Horizontal bar is representative of the Mean ± SEM. *P value < 0.05. Fig. S4. Comparison of the frequencies of CD4+ Foxp3+ subsets in patients with tongue SCC with at least one involved lymph node (LN +) or without lymph node involvement (LN‒). CD4+ T cells gated on the lymphocyte population and then the frequencies of T cell subpopulations were determined within CD4+ T cell gate. Horizontal bar is representative of the Mean ± SEM. *P value < 0.05. Fig. S5. Comparison of the frequencies of CD8+ and its subsets in metastatic (MLNs) and non-metastatic lymph nodes (nMLNs) of patients with tongue SCC. CD8+ T cells gated on the lymphocyte population and then the frequencies of T cell subpopulations were determined within CD8+ T cell gate. Horizontal bar is representative of the Mean ± SEM. *P value < 0.05. Fig. S6. Comparison of CD8+ T cells and its subsets in patients with tongue [file 12865_2022_530_MOESM1_ESM.docx]

Supplementary Material


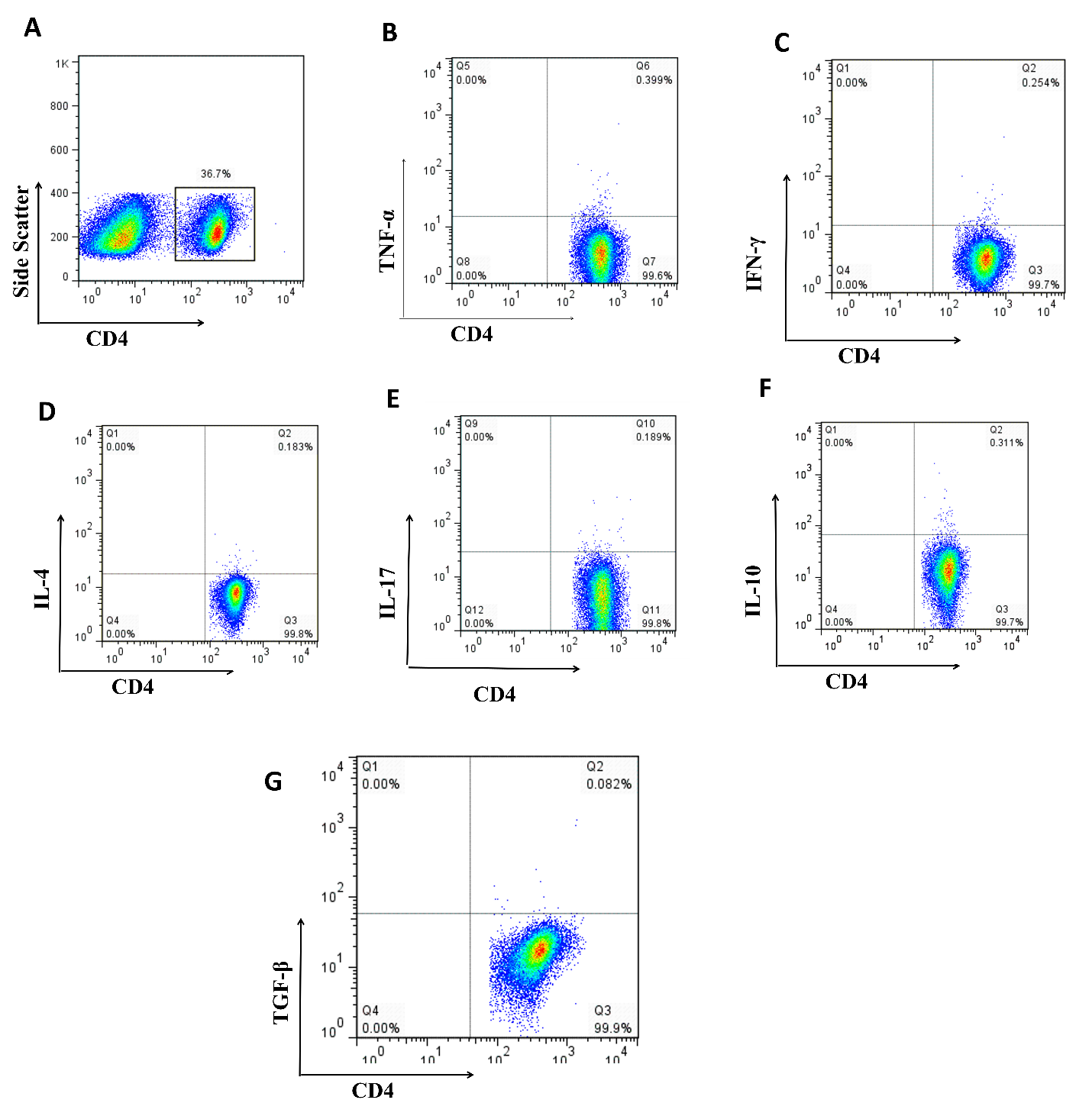


Additional file 1: Fig.S1. Flow cytometry analysis of unstimulated CD4^+^ T cell subsets in the tumor draining lymph nodes of patients with HNSCC. Lymphocytes were gated (A) CD4+ T cells were gated followed by defining these subpopulations in CD4+ T cells gate: (B) TNF-α+ cells (C) IFN-γ + cells (D) IL-4 + cells (E) IL-17 + cells (F) IL-10 + cells (G) TGF-β + cells subsets according to their cognate cytokine expression.


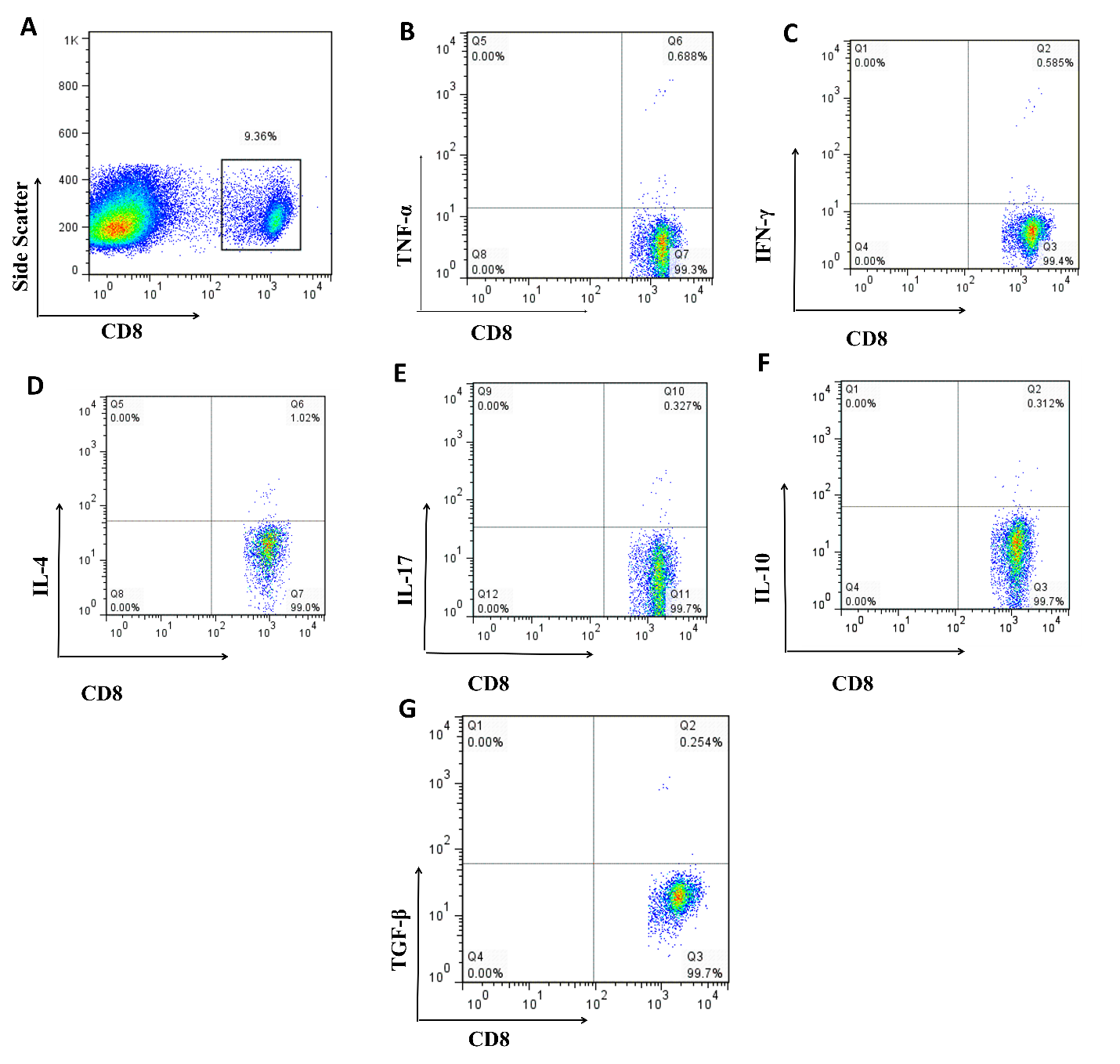


Additional file 1: Fig.S2. Flow cytometry analysis of unstimulated CD8^+^ T cell subsets in the tumor draining lymph nodes of patients with HNSCC. (A) Lymphocytes were gated (B) CD8^+^ T cells were gated followed by defining these subpopulations in CD8^+^ T cells gate: (C) TNF-α^+^ cells (D) IFN-γ ^+^ cells (E) IL-4 ^+^ cells (E) IL-17 ^+^ cells (F) IL-10 ^+^ cells (G) TGF-β ^+^ cells subsets according to their cognate cytokine expression.


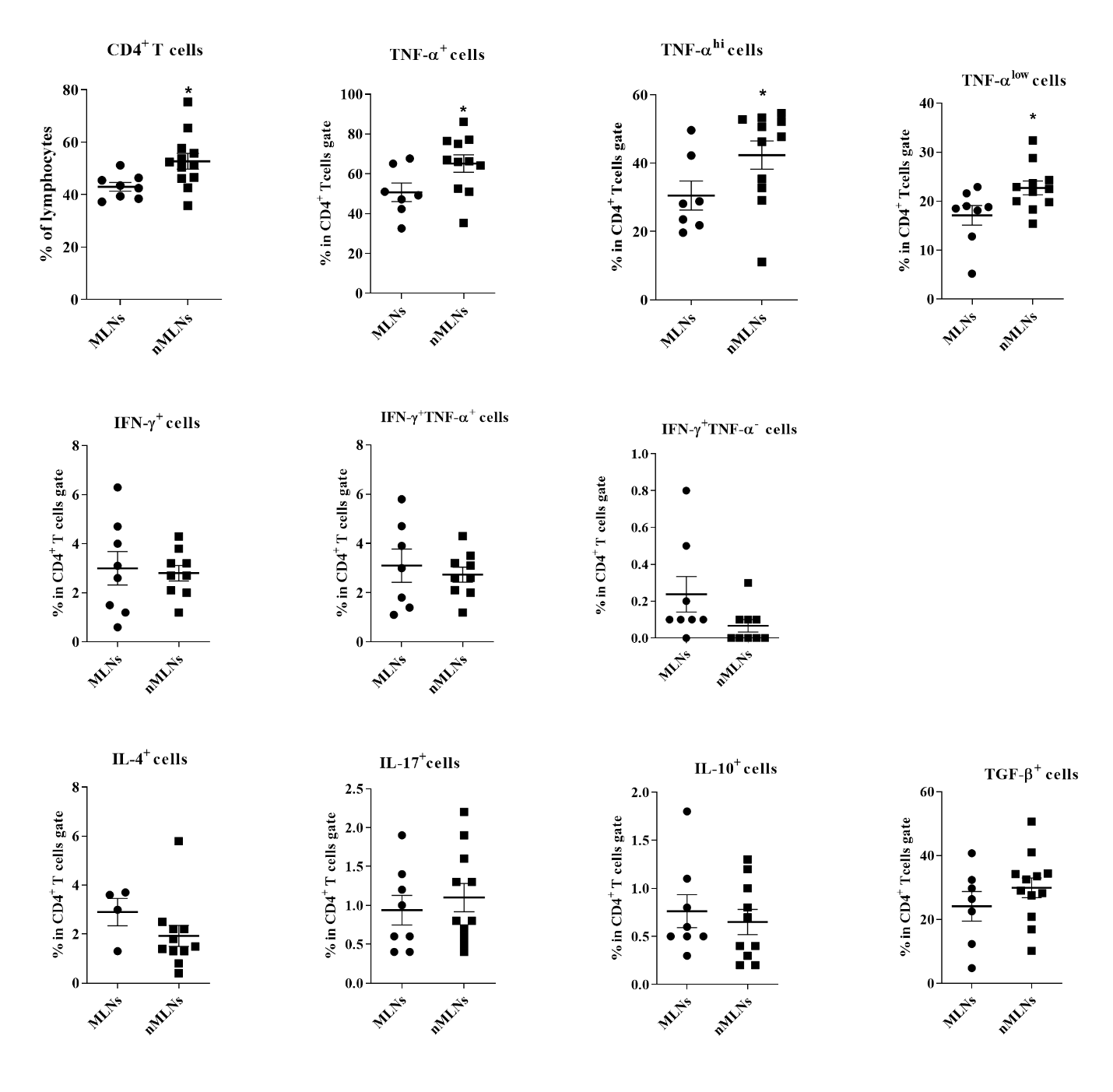
Additional file 1: Fig.S3. Comparison of the frequencies of CD4^+^ and its subsets in metastatic (MLNs) and non-metastatic lymph nodes (nMLNs) of patients with tongue SCC. CD4^+^ T cells gated on the lymphocyte population and then the frequencies of T cell subpopulations were determined within CD4^+^ T cell gate. Horizontal bar is representative of the Mean±SEM. * P value < 0.05.


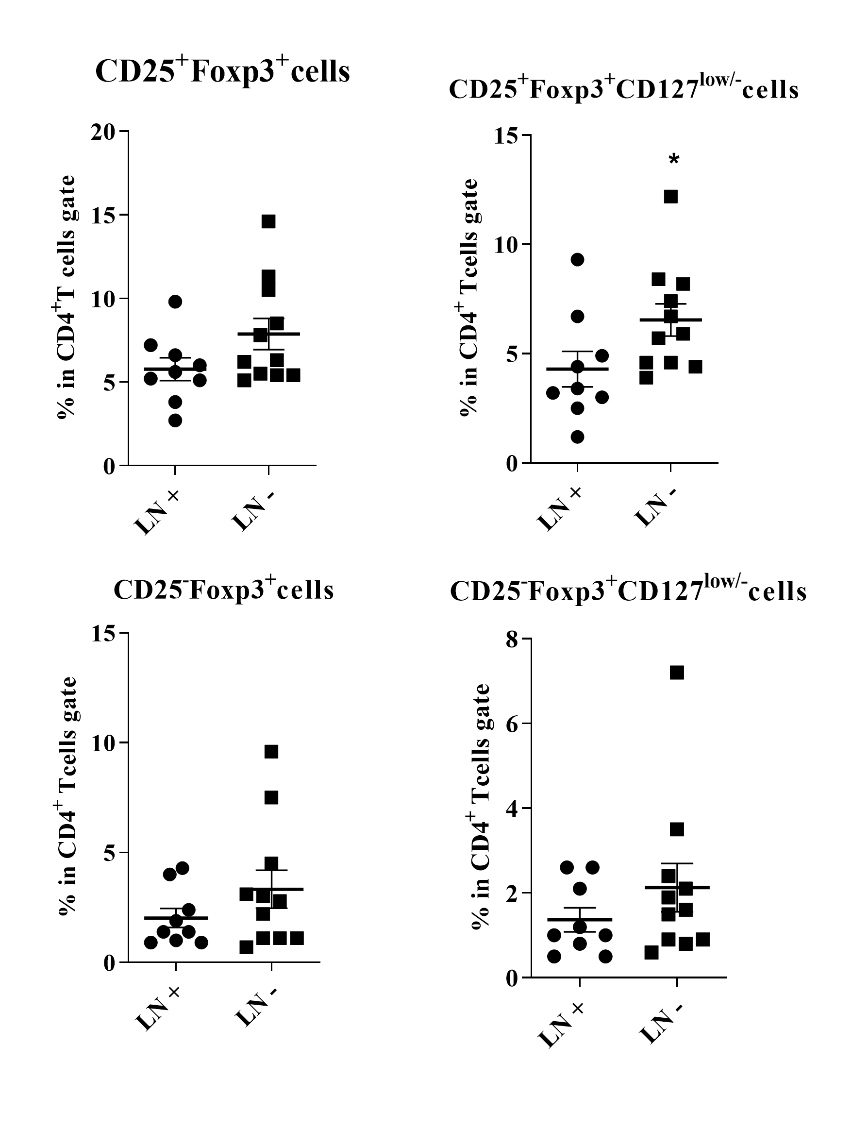


Additional file 1: Fig.S4. Comparison of the frequencies of CD4^+^ Foxp3^+^ subsets in patients with tongue SCC with at least one involved lymph node (LN+) or without lymph node involvement (LN‒). CD4^+^ T cells gated on the lymphocyte population and then the frequencies of T cell subpopulations were determined within CD4^+^ T cell gate. Horizontal bar is representative of the Mean±SEM. * P value < 0.05.


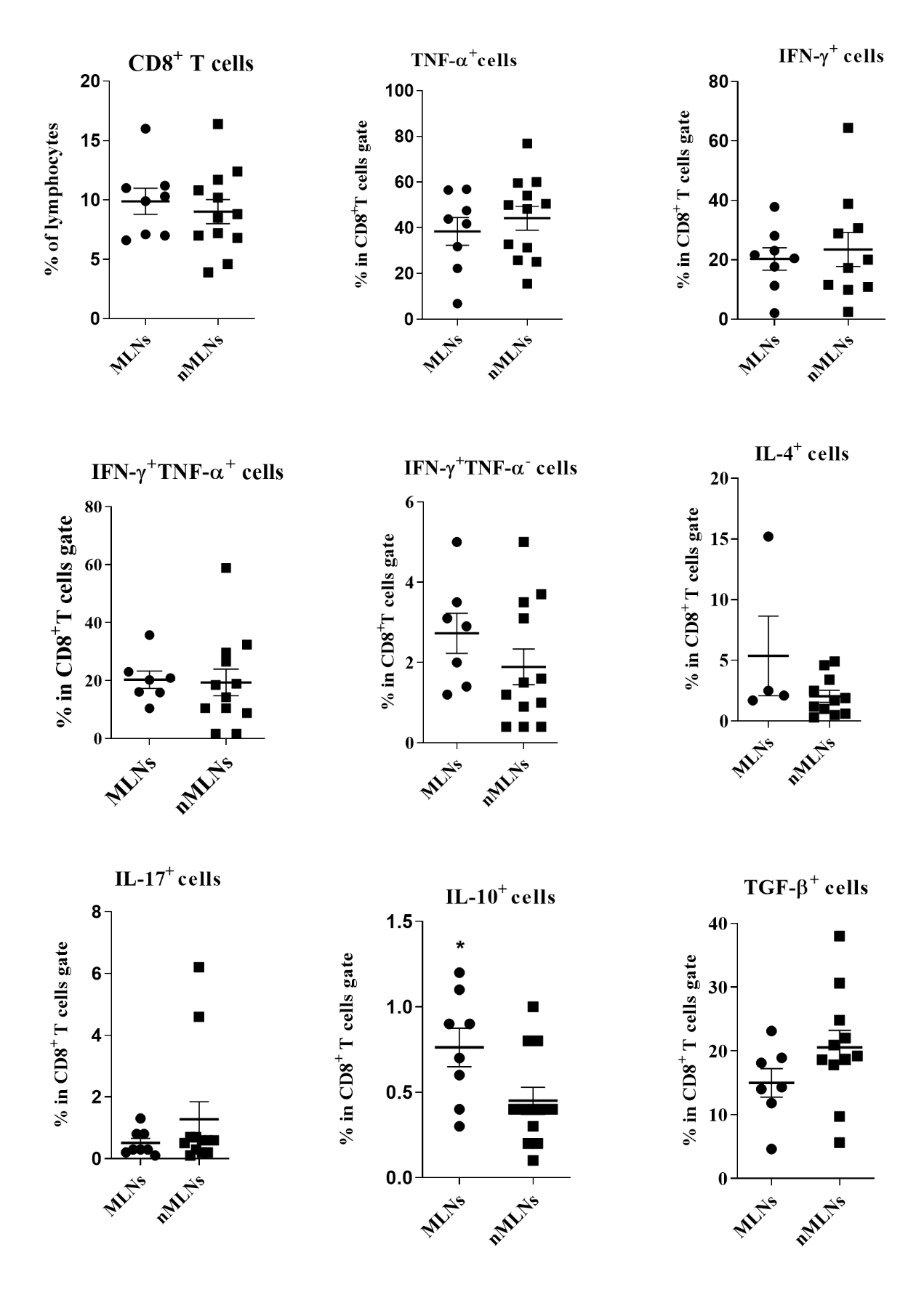


Additional file 1: Fig.S5. Comparison of the frequencies of CD8^+^ and its subsets in metastatic (MLNs) and non-metastatic lymph nodes (nMLNs) of patients with tongue SCC. CD8^+^ T cells gated on the lymphocyte population and then the frequencies of T cell subpopulations were determined within CD8^+^ T cell gate. Horizontal bar is representative of the Mean±SEM. * P value < 0.05.


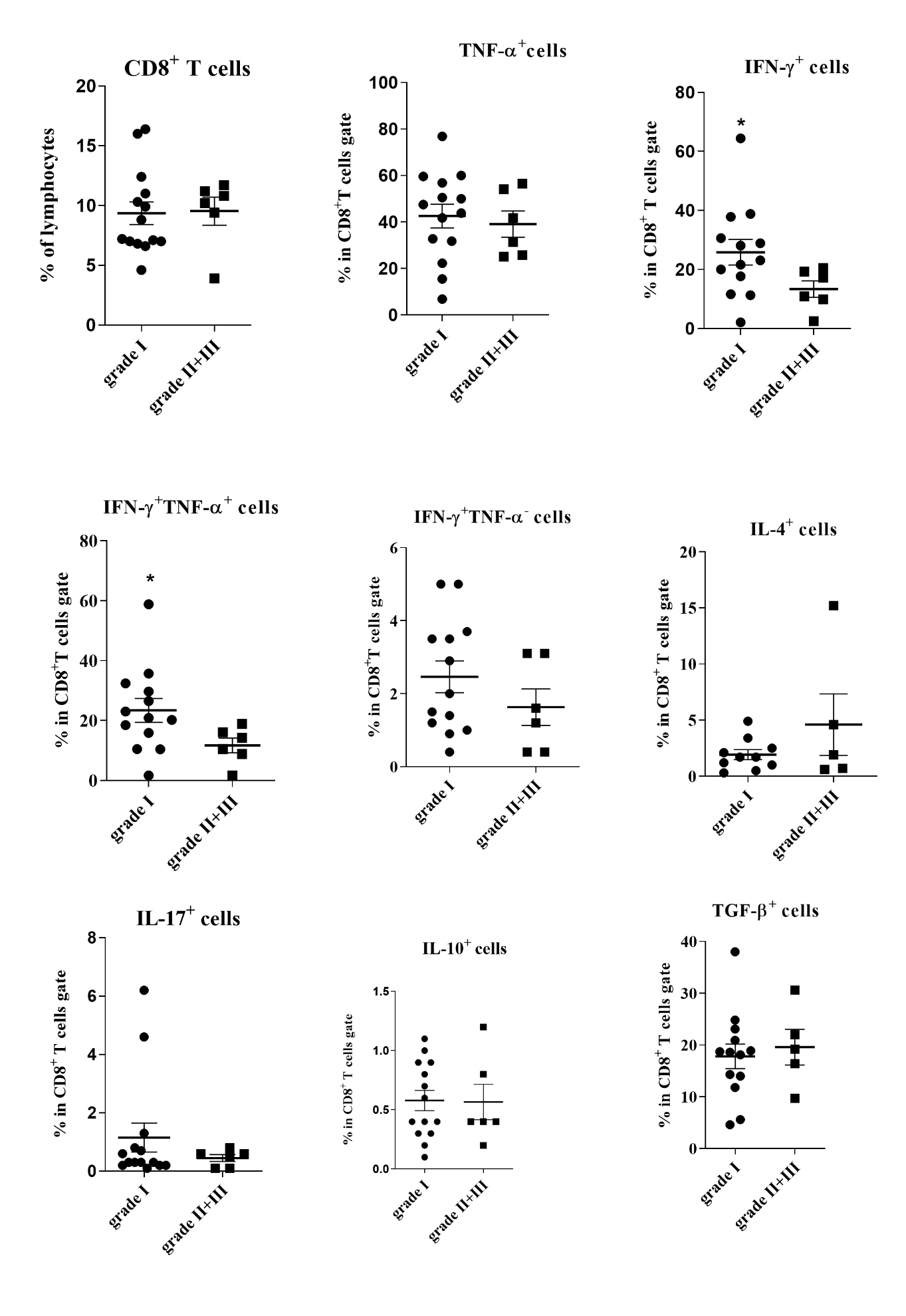


Additional file 1: Fig.S6. Comparison of CD8^+^ T cells and its subsets in patients with tongue SCC with tumor grade I and grade II+III. CD8^+^ T cells gated on the lymphocyte population and then the frequencies of T cell subpopulations were determined within CD8^+^ T cell gate. Horizontal bar is representative of the Mean±SEM, * P value <0.05.


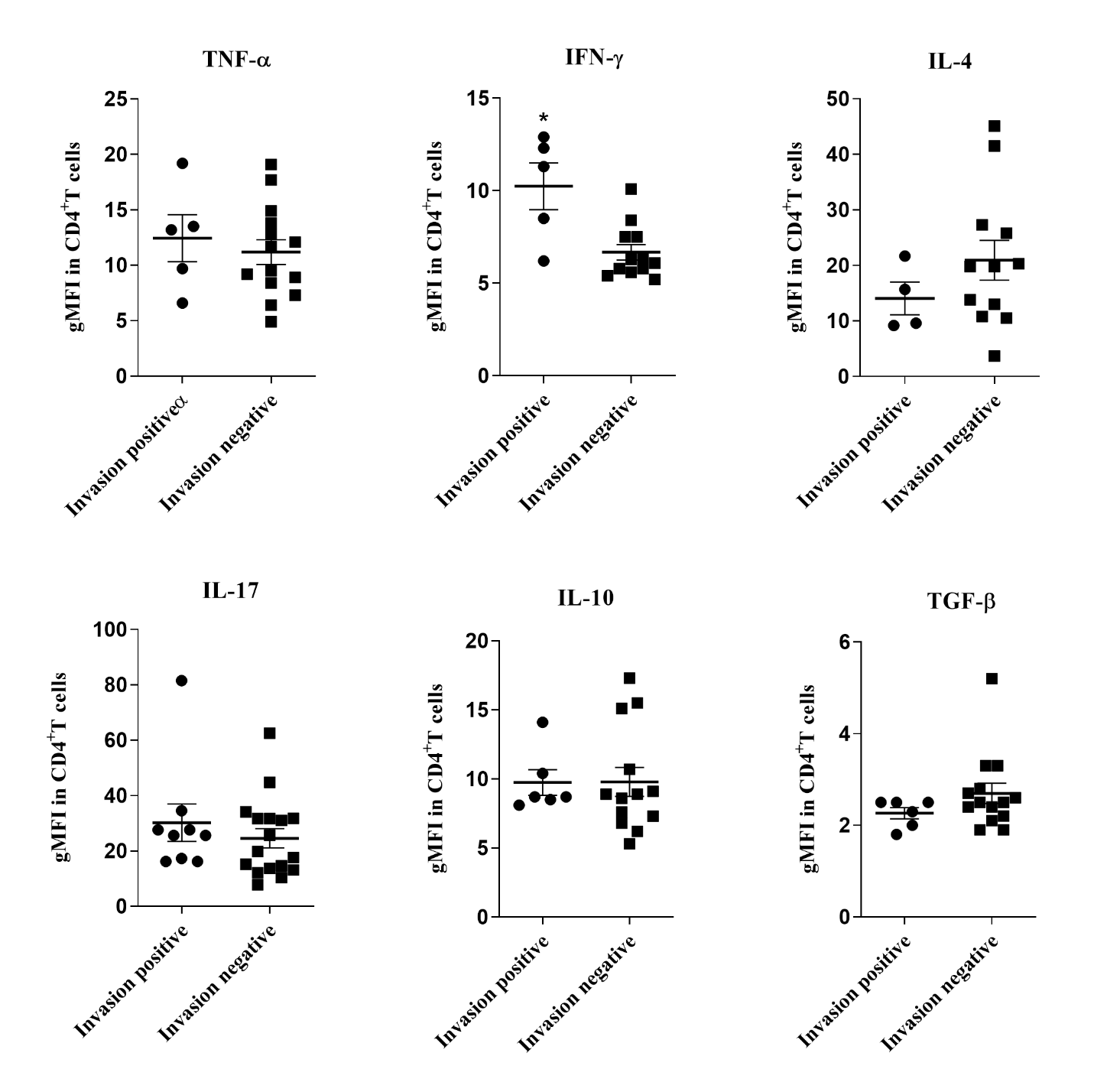


Additional file 1: Fig.S7. Comparison of gMFI of TNF-α, IFN-γ, IL-4, IL-17, IL-10 and TGF-β in CD4^+^ lymphocytes in TDLNs of tongue SCC with or without perineural and/or lymphovascular invasion. Horizontal bar is representative of the Mean±SEM. * P value <0.05.


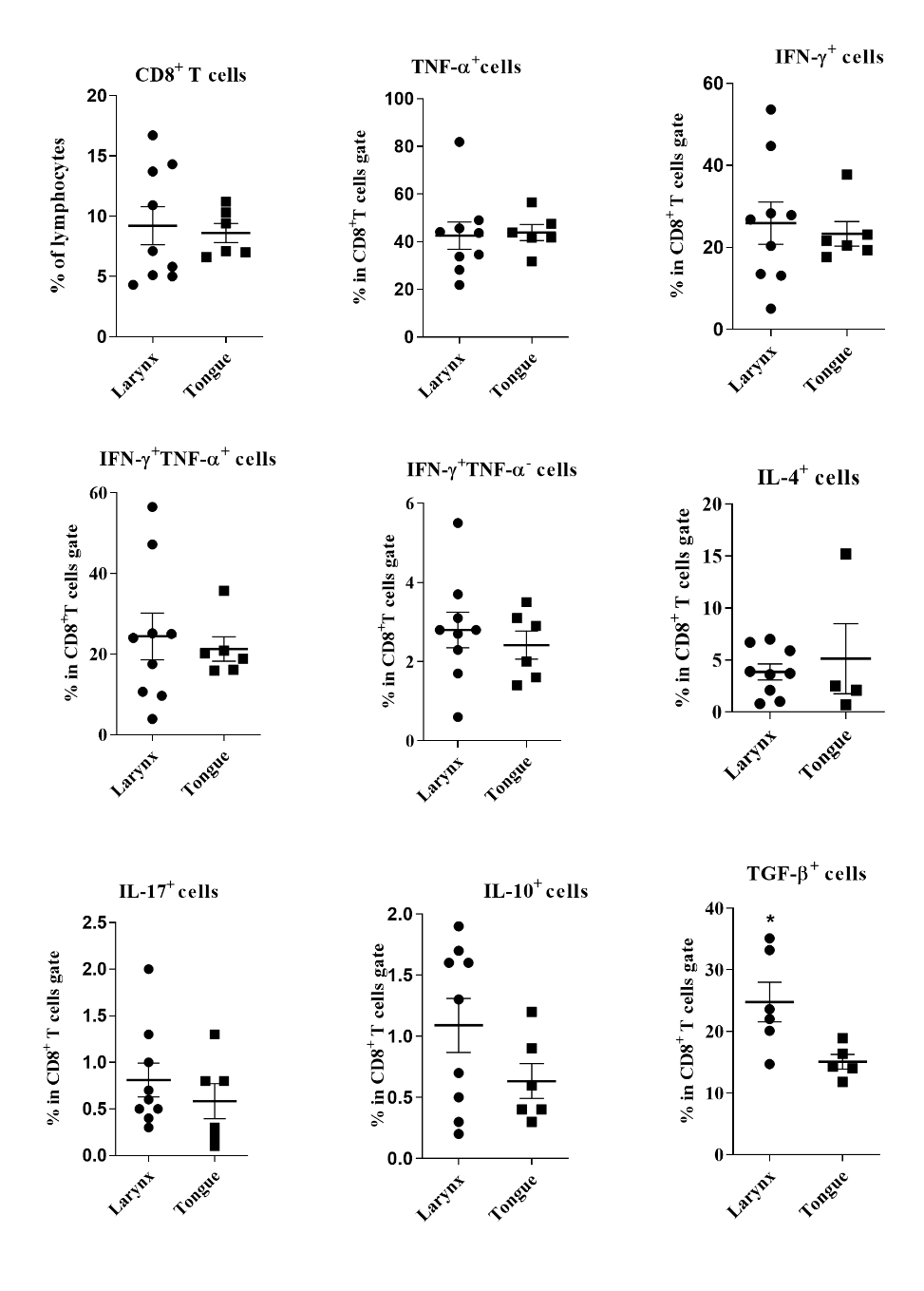


Additional file 1: Fig.S8. Comparison of CD8^+^ T cells and its subsets in TDLNs of tongue and laryngeal SCC with the stage IV of disease. Horizontal bar is representative of the Mean±SEM, * P value < 0.05.


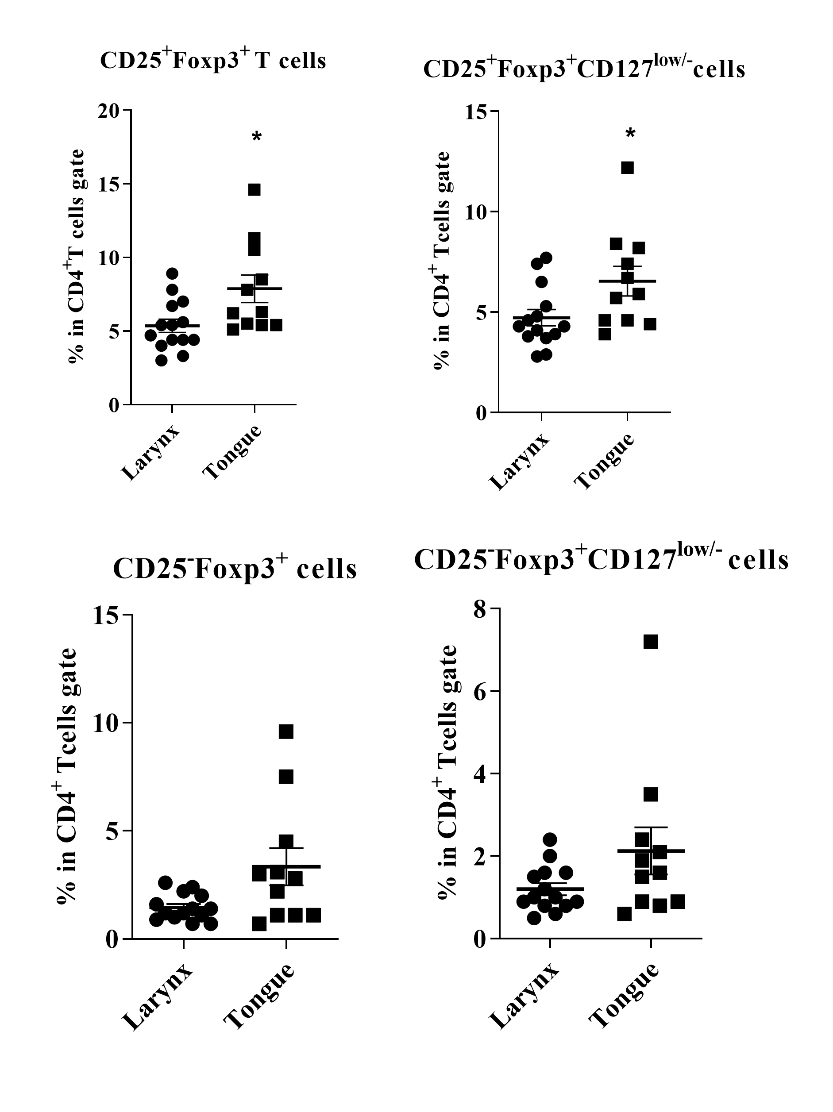


Additional file 1: Fig.S9. Comparison of CD4^+^Foxp3^+^T cells subsets in TDLNs of tongue and laryngeal SCC without LN involvement. Horizontal bar is representative of the Mean±SEM, * P value < 0.05.


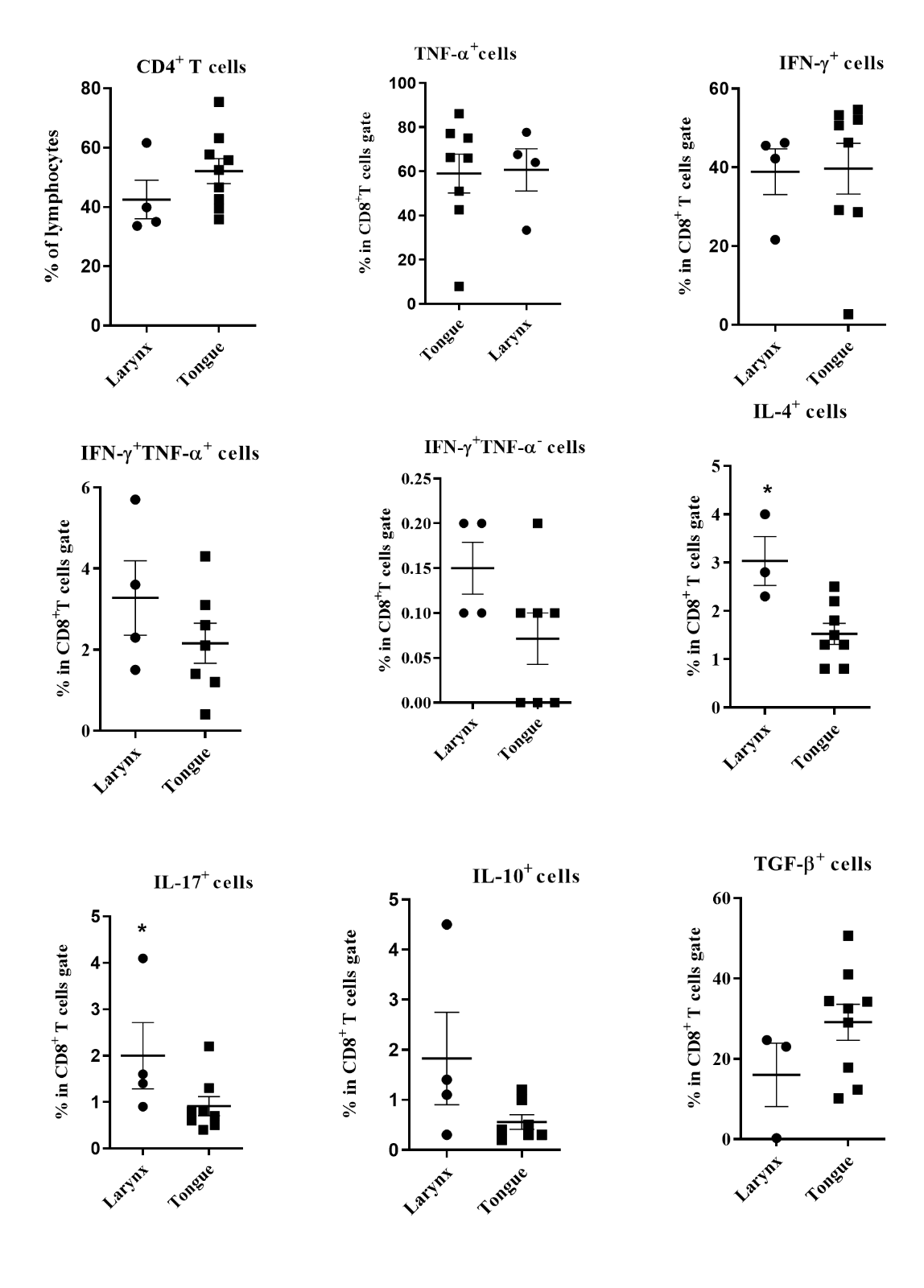


Additional file 1: Fig.S10. Comparison of CD4^+^ T cells subsets in TDLNs of tongue and laryngeal SCC without LN involvement. Horizontal bar is representative of the Mean±SEM, * P value < 0.05, ** P value <0.01.


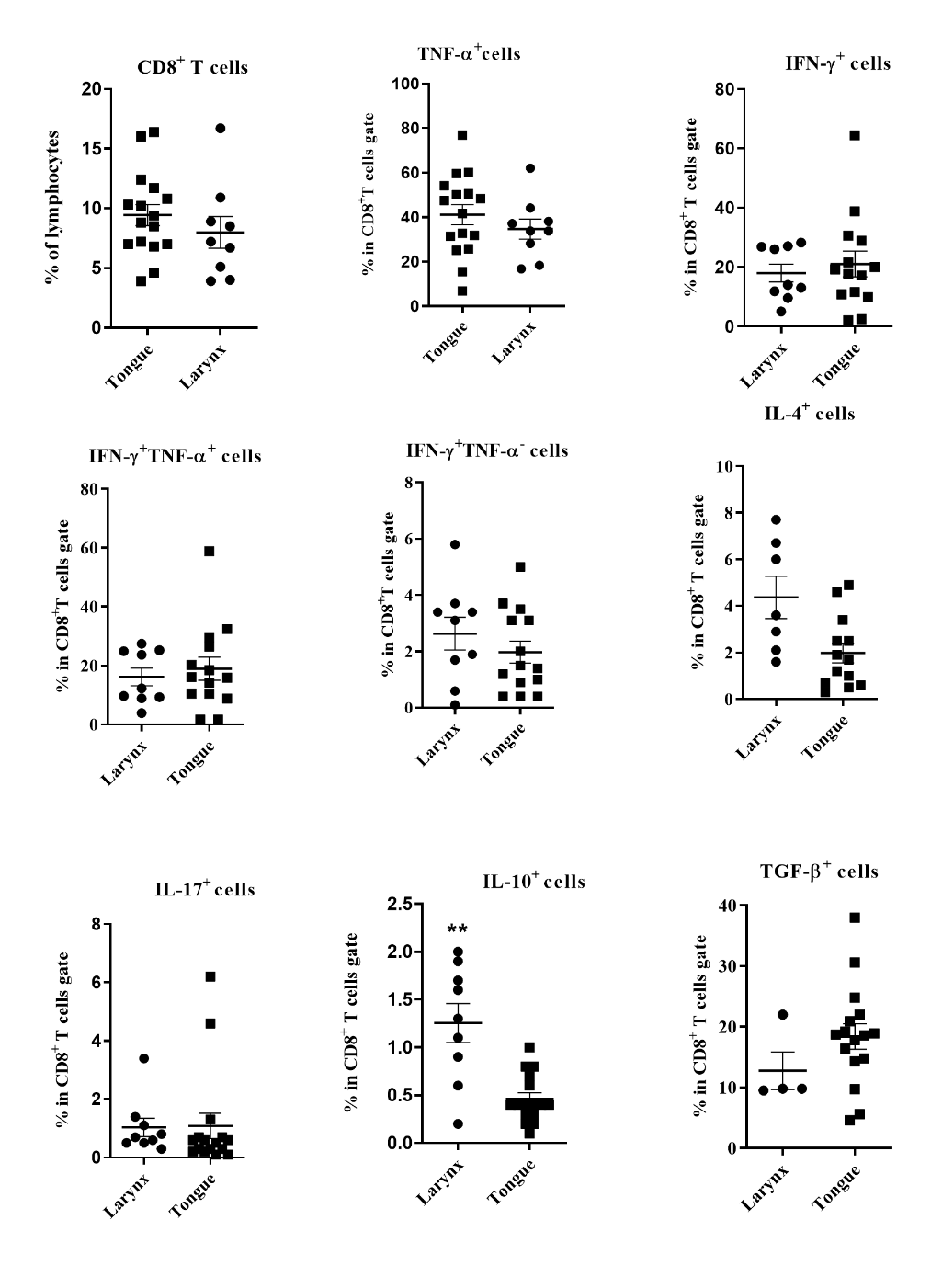


Additional file 1: Fig.S11. Comparison of CD4^+^ T cells and its subsets in TDLNs of tongue and laryngeal SCC with tumor size≤3cm. Horizontal bar is representative of the Mean±SEM, *P value < 0.05


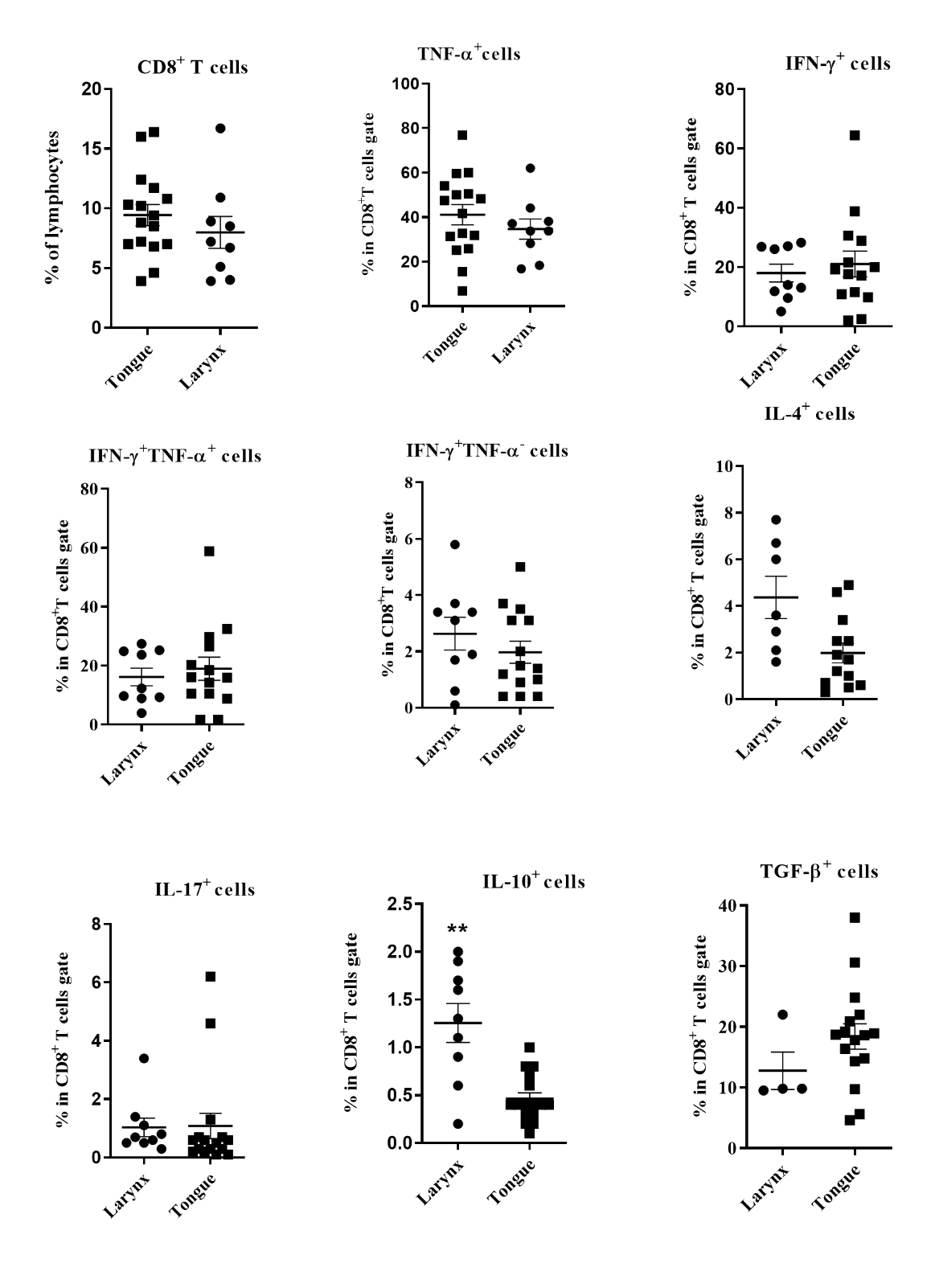


Additional file 1: Fig.S12. Comparison of CD8^+^ T cells and its subsets in TDLNs of tongue and laryngeal SCC with tumor size≤3cm. Horizontal bar is representative of the Mean±SEM, ** P value < 0.01


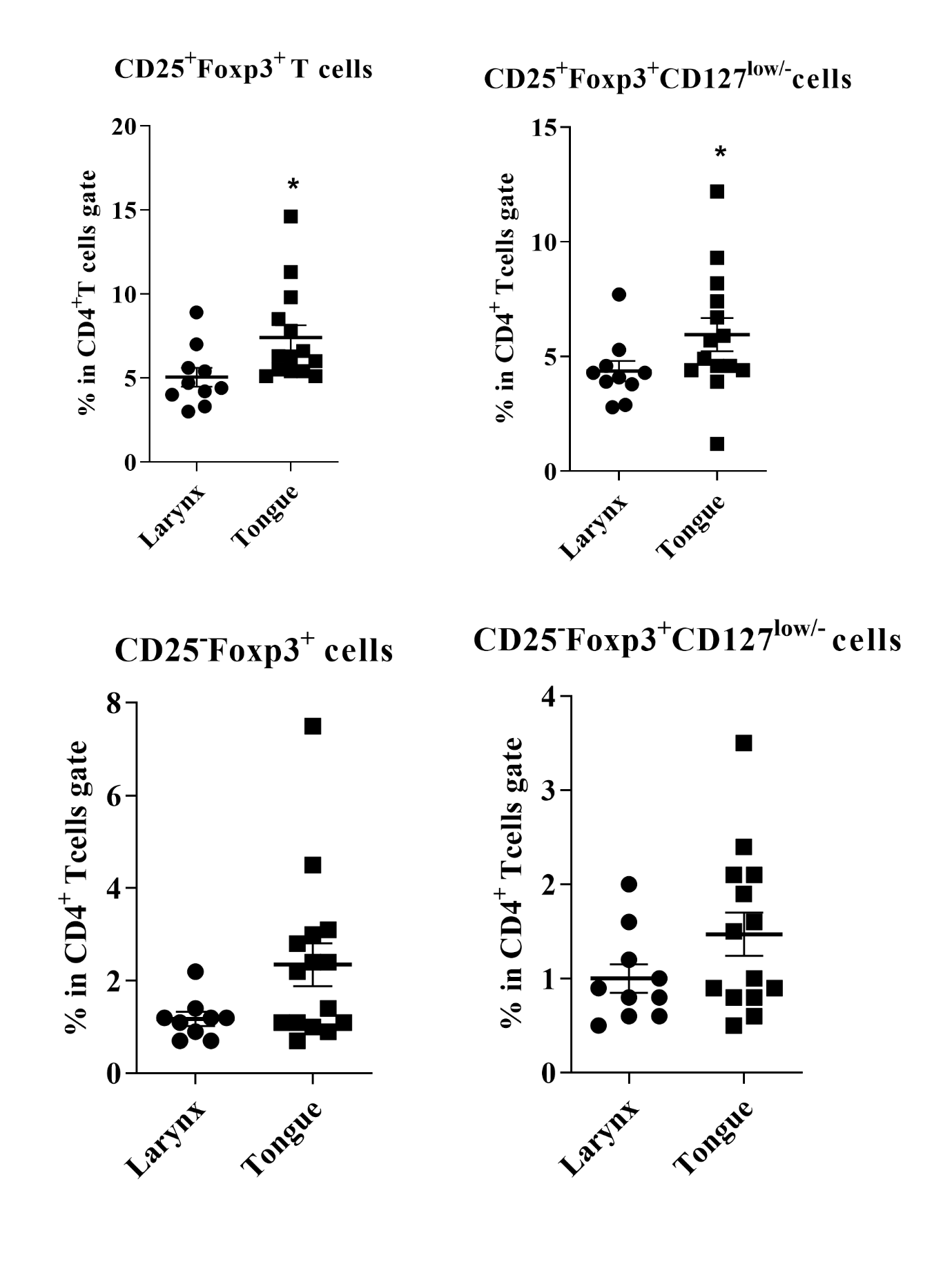
 Additional file 1: Fig.S13. Comparison of CD4^+^Foxp3^+^T cells subsets in TDLNs of tongue and laryngeal SCC with tumor size≤3cm. Horizontal bar is representative of the Mean±SEM, * P value < 0.05
